# Exploring the Mechanism of Yiqi Qingre Ziyin Method in Regulating Neuropeptide Expression for the Treatment of Atrophic Rhinitis

**DOI:** 10.1155/2022/4416637

**Published:** 2022-03-08

**Authors:** Lixing Lu, Xueran Kang, Bin Yi, Chenyan Jiang, Xiaojun Yan, Bin Chen, Yuxing Sun, Fangze Shi, Yuanbo Luo, Yisheng Chen, Runjie Shi

**Affiliations:** ^1^Department of Otorhinolaryngology Head and Neck Surgery, Shanghai Ninth People's Hospital, Shanghai Jiao Tong University School of Medicine, China; ^2^Ear Institute Shanghai Jiaotong University School of Medicine, Shanghai Key Laboratory of Translational Medicine on Ear and Nose Diseases, China; ^3^Department of Sports Medicine, Huashan Hospital, Fudan University, Shanghai, China

## Abstract

Atrophic rhinitis (AR) is a chronic disease that causes severe structural changes to the nasal mucosa leading to squamous epithelial metaplasia. However, treatment regarding AR remains a major challenge. We used network pharmacology and molecular docking methods to explore the potential mechanisms of the Yiqi Qingre Ziyin method to modulate neuropeptides in the treatment of AR. The active ingredients of the Yiqi Qingre Ziyin method and their targets of action were obtained from the Traditional Chinese Medicine Systematic Pharmacology Database Analysis Platform (TCMSP). Disease targets for AR were obtained from four databases: GeneCards, PharmGKB, DrugBank, and Online Mendelian Inheritance in Man (OMIM). A total of 59 active ingredients, 39 potential targets, and 76 relevant neuropeptides were obtained after deduplication. We constructed target interaction networks with the STRING database. Gene Ontology (GO) enrichment analysis and Kyoto Encyclopedia of Genes and Genomes (KEGG) pathway enrichment analysis were performed on the 14 potential target proteins. We used Cytoscape software to construct the “drug-active ingredient-potential target” and “ingredient-target-pathway” networks of the Yiqi Qingre Ziyin method for treating AR. Molecular docking results suggest that dipeptidyl peptidase 4 (*DPP4*), opioid receptor gene d1 (*OPRD1*), and opioid receptor m1 (*OPRM1*) are key targets for the Yiqi Qingre Ziyin method. Therefore, this study proposed a potential mechanism for the treatment of AR by affecting the expression of neuropeptide-related genes (including *DPP4*, *OPRD1*, and *OPRM1*), which may potentially improve the immune microenvironment of the nasal mucosa.

## 1. Introduction

Atrophic rhinitis (AR) is a chronic disease that causes severe structural changes to the nasal mucosa leading to squamous epithelial metaplasia, loss of cupped cells, and occlusion of the vascular cavity [1, 2]. Chronic infections, autoimmune diseases, endocrine disorders, nutritional deficiencies, such as fat-soluble vitamin deficiencies, and hereditary or iron deficiency anemia are suspects as causative agents of this disease. However, a consensus has not been reached [3, 4], leading to unsatisfactory results in modern medical treatment, which is mostly based on conservative treatment with nasal rinses, drops, antibiotics, antibacterial drugs, immunosuppressants, and vasodilators. Our previous studies have found that AR may be associated with septoplasty [5]. Surgical treatments are available to reduce the size, promote regeneration of normal tissue, and improve blood circulation in the nasal cavity. However, the benefits of surgical treatments are uncertain and can be painful. Besides, symptomatic drugs such as immunosuppressive drugs have certain toxic side effects [3]. These factors make AR a progressively difficult disease to treat. The internal conditioning methods of traditional Chinese medicines (TCM) have certain advantages in treating such diseases. Patients are opting for them increasingly [6].

In Chinese medicine, AR, also known as “Bigao,” is commonly associated with the deficiency of the “lung and spleen.” The identification and typing of diseases is the core of diagnosis in the theory of Chinese medicine. The treatment should benefit the Qi, clear heat, and nourish Yin. However, that theory of Chinese medicine was performed at the subjective level, lacking conclusive evidence. In previous studies, scholars found that neurogenic inflammation tests are more specific than traditional eosinophil counts or sIgE tests. And there is a stronger correspondence between these tests and TCM evidence [7–9]. With the development of network pharmacology and structural biology in recent years, a reliable approach has been provided to explore the mechanism of AR progression.

The sympathetic and parasympathetic nervous systems regulate the complex structure of the nasal mucosa. However, it is also controlled by the nonadrenergic noncholinergic nervous system (NANC) [10–12]. NANC mainly consists of sensory nerves like nerve fibers. Generally, the nerve fiber receptors are isolated because of the integrity of the nasal mucosa epithelium. AR disrupts the epithelial integrity and exposes the receptors, resulting in the production of large amounts of sense neuropeptides [13]. Therefore, we hypothesized that neuropeptides may play an important role in the development and progression of AR.

This study explored the mechanism of the Yiqi Qingre Ziyin method for the treatment of AR through network pharmacology and molecular docking.

## 2. Methods

### 2.1. Drug Formulations

Based on the literature review, an herbal prescription was summarized [9]. The prescription consisted of *radix astragali* (20 g), *Poria* (15 g), *Atractylodes macrocephala Koidz* (AMK) (12 g), *angelica* (12 g), *scrophulariae* (12 g), *Ligustrum lucidum Ait* (LIA) (15 g), *kudzu* (12 g), and *red peony root* (RPR) (9 g). *Codonopsis pilosula* (20 g) was added to the prescription when patients presented with significant “qi” deficiency syndrome. In previous research, ingredients and targets of these herbs were downloaded from the traditional Chinese medicine systems pharmacology database [14–17]. An ingredient was defined as active when a combination of the following conditions was true: OB > 30% and DL > 0.18.

### 2.2. Screening of Chinese Herbal Ingredients and Target Proteins and Construction of a Chinese Herbal-Component-Target Visualization Network Map

Drug targets were converted to gene names using the UniProt (https://www.uniprot.org/) database. Proteins related to AR were gathered from four datasets, including GeneCards (https://www.genecards.org/), OMIM (https://omim.org/), PharmGKB (https://www.pharmgkb.org), and DrugBank (https://www.drugbank.com). The aggregation and repetition cut-out work was finished using R software [18]. Then, Cytoscape 3.7.1 was used to construct the herbal-component-target visualization network diagram [19].

### 2.3. Extraction of Key Targets and Construction of Protein-Protein Interaction Networks

Potential targets were imported into the STRING 11.0 database (https://string-db.org/) as previous researches [20, 21]. Species were set to *Homo sapiens*. Protein interaction relationships were obtained using a filter with a combined score ≥ 0.7 and removing free nodes. Download the result file in tsv format, and import it into Cytoscape 3.7.1. Use the network analyzer in this software to perform network topology analysis and build a PPI network [22].

### 2.4. Enrichment Analysis of Pathways

Enrichment analysis was performed for the 14 proteins screened using the Kyoto Encyclopedia of Genes and Genomes (KEGG) and Gene Ontology (GO). The top-ranked genes and those with *P* values less than 0.05 were visualized with R software as previous researches [23–26].

### 2.5. Molecular Docking Analysis

The molecular docking software AUTODOCK 4 was used to validate the molecular docking of the target with the highest correspondence value for each herbal medicine in the PPI network [27]. The RCSB PDB database (http://www.rcsb.org/) and the ZINC database (http://zinc.docking.org/) were used to obtain the structures of target proteins and ligands. Docking results are presented in the form of binding energy, smaller scores indicating more stable binding [28, 29].

### 2.6. Molecular Dynamics Simulation (MDS)

The complex system of compound and target protein obtained by molecular docking was used as the initial structure for all-atom molecular dynamics simulations (MDS). Create small molecule force fields using ACPYPE Server [30, 31]. The protein force field is described using CHARMM36 [32]. The truncated tetrahedral TIP3P was added as a solvent cartridge in the system. In this case, Na+/Cl- is used to balance the system charge.

GROMACS software was used in this study to perform the system simulations [33, 34]. Energy optimisation is carried out first. The temperature of the system is then slowly increased from 0 K to 307 K at a fixed volume and a constant rate of temperature increase. To further homogenize the distribution of solvent molecules in the solvent box, the NVT and NPT simulations were run in order at a system maintenance temperature of 307 K. Finally, to perform molecular dynamics simulations, the composite system was simulated under periodic boundary conditions with a system pressure of 1 atm, a 2 fs integration step, and trajectories stored at 1 ps intervals.

## 3. Results

### 3.1. Screening of Active Ingredients for Yiqi Qingre Ziyin Method

**Figure 1:**
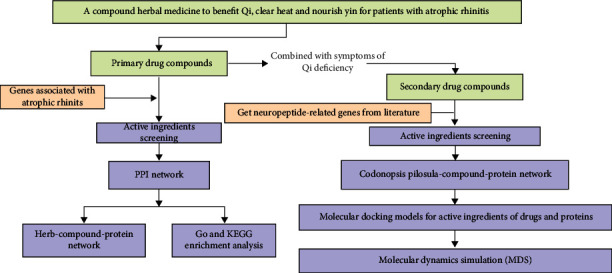
Flowchart of the network pharmacology study of Yiqi Qingre Ziyin method in the treatment of AR.

**Figure 2:**
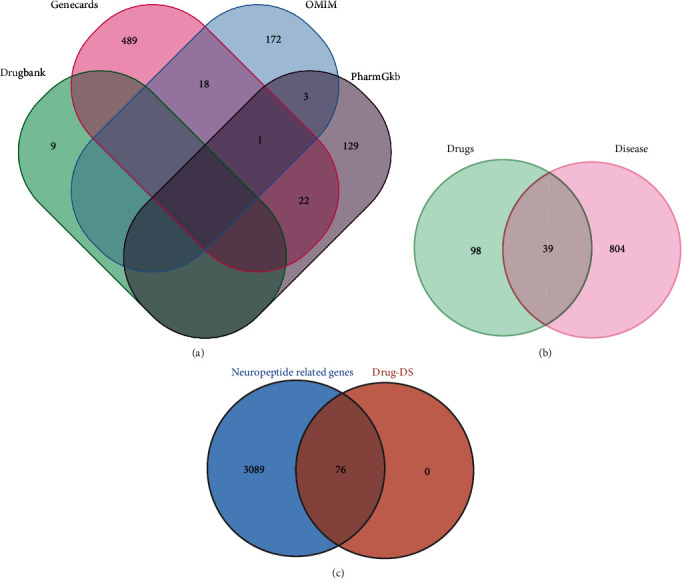
Extraction of key proteins associated with disease. (a) All genes associated with AR. (b) Thirty-nine AR-associated genes intersecting with the effective ingredient target genes of the Yiqi Qingre Ziyin method. (c) All the neuropeptide-related proteins and those 76 targeted by active ingredients of *Codonopsis pilosula* in the Yiqi Qingre Ziyin method.

The research flowchart for this study is shown in Figure 1. We first obtained the active ingredients of the Yiqi Qingre Ziyin method. The proteins corresponding to 147 genes were found to be potential targets for the active ingredients of this method. The proteins corresponding to 76 genes were found to be potential targets of *Codonopsis pilosula*. A total of 843 disease-associated genes were screened (Figure 2(a)), and 39 genes were identified as potential targets (Figure 2(b)). All targets of *Codonopsis pilosula*, also named as Dangshen (DS), were found to be associated with neuropeptide-related genes (Figure 2(c)).

### 3.2. Construction of Component-Target Gene Network and Protein-Protein Interaction (PPI) Networks

**Figure 3:**
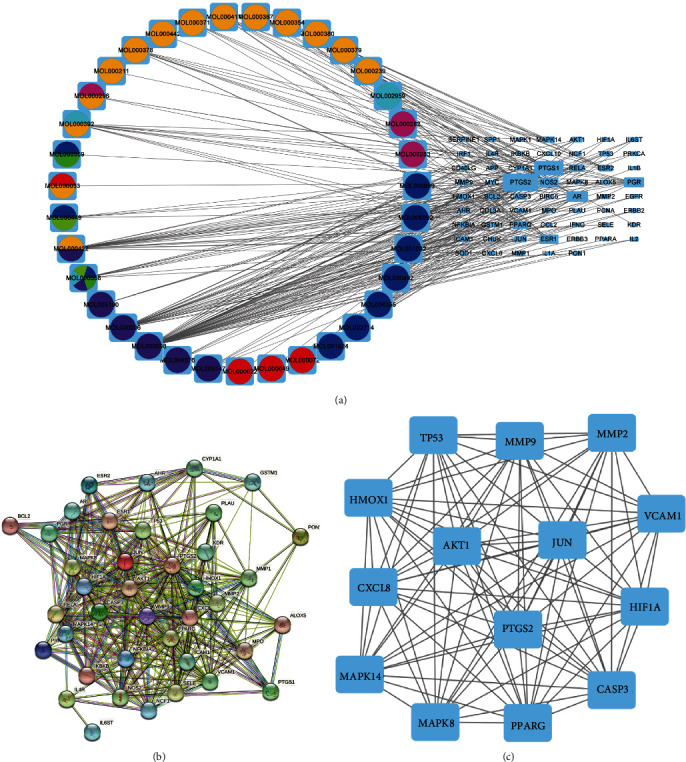
The Yiqi Qingre Ziyin method's “drug-active ingredient-potential target” network and PPI network for the treatment of AR. (a) Yiqi Qingre Ziyin method's “drug-active ingredient-potential target” network for the treatment of AR. (b, c) Yiqi Qingre Ziyin method PPI network for treating AR.

For regular AR patients, Yiqi Qingre Ziyin herbal soup is often given orally in clinical practice. A disease-drug-component-potential-target network map was constructed using Cytoscape (Figure 3(a)). As shown in the figure, red, blue, pink, cyan, yellow, green, and reseda indicate Atractylodes macrocephala Koidz (AMK), red peony root (RPR), Poria, kudzu, radix astragali, scrophulariae, and angelica, respectively (Figure 3(a)). Besides, these genes were imported into STRING to build the PPI network (Figure 3(b)). Based on this data, the network topology analysis was performed using Cytoscape 3.7.1 [35]. The network has 14 nodes and 89 edges. A total of 14 key genes were acquired: *AKT1*, *CASP3*, *CXCL8*, *HIF1A*, *HMOX1*, *JUN*, *MAPK14*, *MAPK8*, *MMP2*, *MMP9*, *PPARG*, *PTGS2*, *TP53*, and *VCAM1* (Figure 3(c)).

### 3.3. GO and KEGG Enrichment Analysis

**Figure 4:**
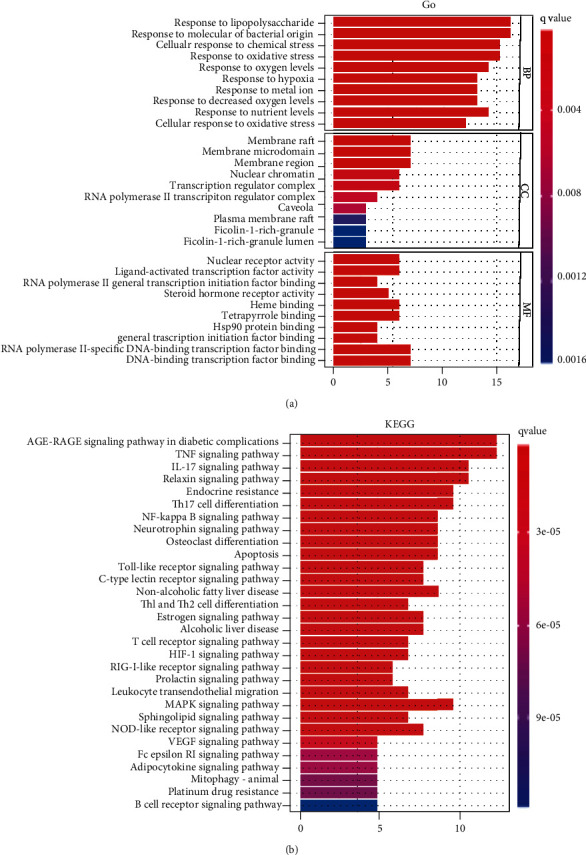
(a) GO and (b) KEGG enrichment analysis of potential targets for the treatment of AR by the Yiqi Qingre Ziyin method.

Fourteen key genes (*AKT1*, *CASP3*, *CXCL8*, *HIF1A*, *HMOX1*, *JUN*, *MAPK14*, *MAPK8*, *MMP2*, *MMP9*, *PPARG*, *PTGS2*, *TP53*, and *VCAM1*) were used for enrichment analysis. The enriched biological processes are responses to lipopolysaccharide, molecules of bacterial origin, chemical stress (cellular response), oxygen levels, hypoxia, metal ion, nutrient levels, and oxidative stress (cellular response). The enriched cellular components are membrane raft, membrane microdomain, membrane region, nuclear chromatin, transcription regulator complex, RNA polymerase II, caveola, plasma membrane raft, ficolin-1-rich granule, and ficolin-1-rich granule lumen. The enriched molecular functions are nuclear receptor activity, ligand-activated transcription factor activity, RNA polymerase II general transcription initiation factor binding, steroid hormone receptor activity, heme binding, tetrapyrrole binding, Hsp90 protein binding, general transcription initiation factor binding, RNA polymerase II-specific DNA-binding transcription factor binding, and DNA-binding transcription factor binding. The above results suggest that the Yiqi Qingre Ziyin method presents a multitarget and multipathway action for the treatment of AR (Figure 4(a)) (Supplementary Table 1). In the KEGG metabolic pathway enrichment, a total of 78 relevant metabolic pathways were obtained at *P* < 0.05. The key KEGG enrichment pathway maps were extracted and visualized in descending order of *P* (Figure 4(b)) (Supplementary Table 2). The main pathways of the Yiqi Qingre Ziyin method for the treatment of AR are AGE-RAGE in diabetic complications, TNF, IL-17, relaxin, endocrine resistance, Th17 cell differentiation, NF-kappa B, neurotrophin, osteoclast differentiation, apoptosis, Toll-like receptor, C-type lectin receptor, nonalcoholic fatty liver disease, Th1 and Th2 cell differentiation, estrogen, alcoholic liver disease, T cell receptor, HIF-1, RIG-I-like receptor, prolactin, leukocyte transendothelial migration, MAPK, sphingolipid, NOD-like receptor, VEGF, Fc epsilon RI, adipocytokine, mitophagy–animal, platinum drug resistance, and B cell receptor.

### 3.4. Analysis of Neuropeptide-Related Target Gene-Pathway Networks and Molecular Docking Results

**Figure 5:**
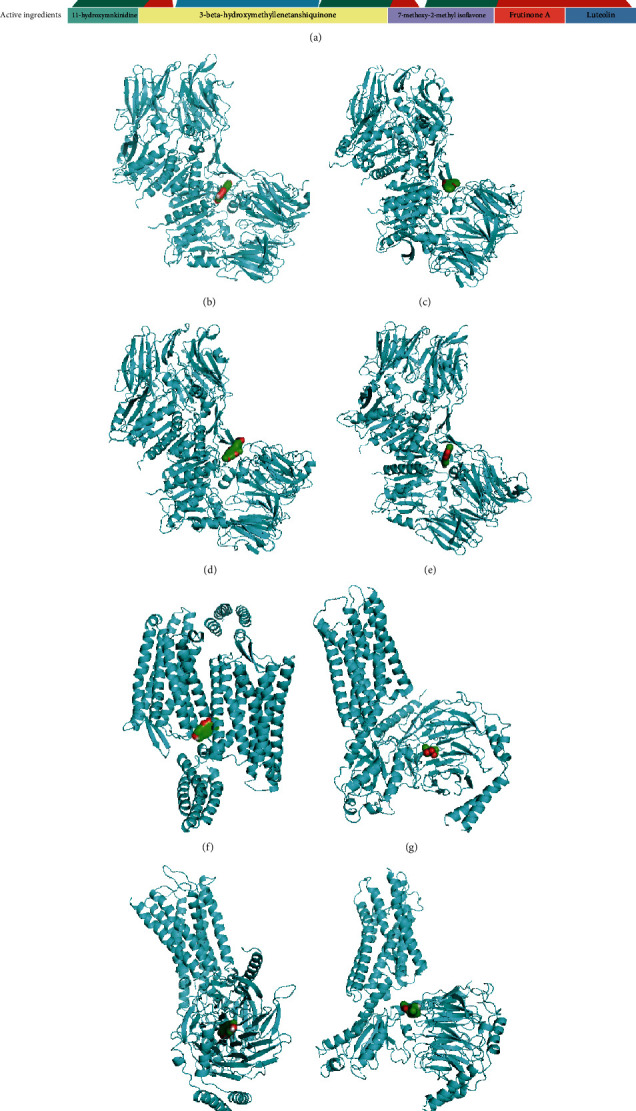
Docking pattern of potential therapeutic targets of *Codonopsis pilosula* herbal components from Yiqi Qingre Ziyin method. (a) Curving diagram of the interactions between potential therapeutic targets of this method and components of *Codonopsis pilosula*; (b) luteolin; (c) frutinone A; (d) 7-methoxy-2-methyl isoflavone; (e) 3-*beta*-hydroxymethyllenetanshiquinone to DPP4; (f) 3-*beta*-hydroxymethyllenetanshiquinone to OPRD1; (g) 7-methoxy-2-methyl isoflavone; (h) 11-hydroxyrankinidine; (i) 3-*beta*-hydroxymethyllenetanshiquinone to OPRM1.

*Codonopsis pilosula* was added to the prescription when patients presented with significant “qi” deficiency syndrome. Of the 76 targeted neuropeptide genes in *Codonopsis pilosula* (Figure 2(c)), *DPP4*, *OPRD1*, and *OPRM1* are the target genes for AR (Figure 5(a)). Luteolin, frutinone A, 7-methoxy-2-methylisoflavone, 3-*beta*-hydroxymethyllenetanshiquinone, and 11-hydroxyrankinidine are effective components of *Codonopsis pilosula* and may have a potential role in AR therapy (Figures 5(b)–5(i)) (Table 1). Drug components (including luteolin, frutinone A, 7-methoxy-2-methyl isoflavone, and 3-*beta*-hydroxymethyllenetanshiquinone) from DS bound to *DPP4* with binding energies of -7.6 kcal/mol, -8.6 kcal/mol, -7.8 kcal/mol, and -8.9 kcal/mol, respectively. 7-Methoxy-2-methylisoflavone, 3-*beta*-hydroxymethyllenetanshiquinone, and 11-hydroxyrankinidine bound to *OPRM1* with binding energies of -8.9 kcal/mol, -8.9 kcal/mol, and -7.4 kcal/mol, respectively. 3-*beta*-Hydroxymethyllenetanshiquinone also binds to *OPRD1* with a binding energy of -8.3 kcal/mol. It is generally accepted that a docking fraction below -4.5 kcal/mol is a binding possibility, and a docking fraction below -6 kcal/mol is strong binding power. The binding docking fractions in this analysis were all below -6 kcal/mol, indicating stable and strong binding. This suggests that the effective components in *Codonopsis pilosula* have a high binding capacity towards neuropeptide-related proteins *DPP4*, *OPRD1*, and *OPRM1*.

### 3.5. Molecular Dynamics Simulations Indicate That the Tiny Chemical 3-*beta*-Hydroxymethyllenetanshiquinone Binds to DPP4 in a Stable Manner

**Figure 6:**
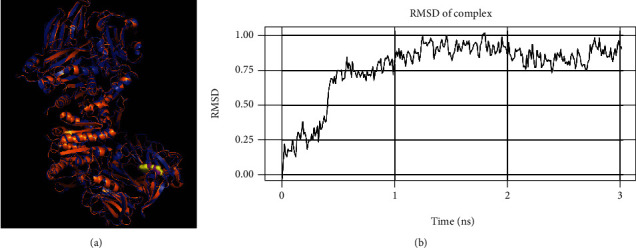
Molecular dynamics simulation (MDS) showing the drug's active component, 3-*beta*-hydroxymethylethylenediamine, binding to DPP4. (a) Protein conformation before and after MDS, with purple indicating pre-MDS conformation and orange indicating post-MDS conformation. (b) During MDS, the variations of 3-*beta*-hydroxymethylethylenediamine and DPP4 in the system are represented as root mean square deviation (RMSD).

The small chemical 3-*beta*-hydroxymethyllenetanshiquinone was shown to have the maximum binding stability to DPP4 in the current anticipated results. The purple color indicates the pre-MDS conformation, and the orange color indicates the post-MDS conformation. It can be seen that both molecules before and after MDS maintain the same binding site (Figure 6(a)). 3-*beta*-Hydroxymethyllenetanshiquinone and DPP4 binding sites were virtually unaltered over MDS, showing that 3-*beta*-hydroxymethyllenetanshiquinone was always securely bound inside the active pocket (Figure 6(b)). And, after MDS, the small molecule seems to be closer to the protein, indicating that 3-*beta*-hydroxymethyllenetanshiquinone can bind to DPP4 at this position in a stable manner. As a result, we hypothesize that 3-*beta*-hydroxymethyllenetanshiquinone, the active component in the Yiqi Qingre Ziyin method, can suppress the development of AR by specifically targeting DPP4.

## 4. Discussion

The early symptoms of primary AR are mild with no obvious dysfunction. Chinese medicine believes that AR is caused by “wind,” “phlegm,” and “dampness.” Patients present with nasal congestion and large amounts of purulent discharge. This leads to a high rate of missed and delayed diagnoses. The etiology of this disease is complex; however, it is generally believed to be related to bacterial infections, plant nervous disorder, and vitamin B2, vitamin A, and vitamin D deficiency [36]. The disease occurs in adolescence with higher incidences in women and gets aggravated during menstruation. Some scholars believe that this disease is related to endocrine immune dysfunction. Considering the complex etiology of the disease, clinical treatment is often symptomatic. However, the therapeutic efficacy is poor.

In the present study, new neuropeptide-related target gene-pathway networks were proposed using network pharmacology and molecular docking techniques. Through MDS research, the active component 3-*beta*-hydroxymethyllenetanshiquinone in Yiqi Qingre Ziyin method was demonstrated to suppress the development of AR by target binding to DPP4.

Luteolin, frutinone A, 7-methoxy-2-methylisoflavone, 3-*beta*-hydroxymethyllenetanshiquinone, and 11-hydroxyrankinidine may serve as key players in the treatment of AR. These drug components have been found in previous studies to play a key role in several diseases. Luteolin has many properties, including antioxidant, antimicrobial, anti-inflammatory, chemopreventive, chemotherapeutic, cardioprotective, antidiabetic, antidiabetic neuroprotective, and antiallergic effects [37–41]. Luteolin inhibits interleukin- (IL-) 1*β*, IL-2, IL-6, IL-8, IL-12, IL-17, TNF-*α*, interferon- (IFN-) *β*, and granulocyte-macrophage colony-stimulating factor. It also increases the levels of IL-10, an anti-inflammatory cytokine. 7-Methoxy-2-methylisoflavone significantly reduces depression in patients, and this antidepressant affects both menopausal women and men [42–44]. GSEA analysis suggests that the Yiqi Qingre Ziyin method targets the IL-17 signaling pathway, Th17 cell differentiation, which suggests that the Yiqi Qingre Ziyin method may regulate the expression of *DPP4* in Th17 cells, thereby alleviating the symptoms of AR.

In this study, *DPP4* was found to be associated with AR. It is also a target gene of *Codonopsis pilosula*. Indeed, as a dipeptidyl peptidase, *DPP4* could cleave dipeptides from the n-terminus of dipeptides, including enterotrophic hormone, neuropeptides, and some chemokines. *DPP4* was found abundantly expressed in IL-17-producing CD4 T cells (Th17 cells) [45, 46]. *DPP4* is an enzymatically active molecule expressed by Th17 cells and has a biologically active enzymatic role that may contribute to the regulation of inflammatory T cell chemotaxis [46]. Studies suggest that *DPP4*-mediated modifications of enzymatic chemokines may regulate T cell migration. Therefore, upregulation of *DPP4* is also associated with disease activity in human autoimmune diseases, such as rheumatoid arthritis and multiple sclerosis [47–50]. In the present study, we verified for the first time that the active ingredient 3-*beta*-hydroxymethyllenetanshiquinone stably binds to DPP4 protein in a computer-simulated system by MDS technique. This suggests that 3-*beta*-hydroxymethyllenetanshiquinone, the active ingredient in Yiqi Qingre Ziyin method, may be able to inhibit the progression of AR by targeting binding to DPP4.

The m-opioid receptor is encoded by the opioid receptor m1 (*OPRM1*) gene and is the primary site of action for the most used opioids. The delta-opioid receptor (DOR) is encoded by the opioid receptor gene (*OPRD1*). Opioid receptors are the targets of a large number of drugs, including heroin and many analgesics. Genetic diversity and DOR expression levels of *OPRD1* are associated with a variety of diseases, including substance abuse and addiction, anorexia nervosa (AN), obesity, and Alzheimer's disease (AD) [51–53]. Under normal conditions, DOR is localized within the cell. Inflammation can cause receptors on dorsal spinal cord neurons to migrate to the membrane surface, where they can be agonized by delta opioid agonists [54–56]. However, further studies on the role of *OPRD1* and *OPRM1* in AR are needed, and this study found that they may serve as potential targets for the Yiqi Qingre Ziyin method in the treatment of AR.

Network pharmacology and molecular docking were used to explore the potential mechanisms of Yiqi Qingre Ziyin method-mediated neurogenic peptides for the treatment of AR. Three neuropeptide-related proteins, *DPP4*, *OPRD1*, and *OPRM1*, were found to be potentially associated with the development of AR. Nonetheless, the particular mechanism of these proteins' action on AR has to be explored further. The active ingredients luteolin, frutinone A, 7-methoxy-2-methylisoflavone, 3-*beta*-hydroxymethyllenetanshiquinone, and 11-hydroxylancidin from *Codonopsis pilosula* bind with *DPP4*, *OPRM1*, and *OPRD1*. These could be key targets for the Yiqi Qingre Ziyin method. The results of this study suggest that the Yiqi Qingre Ziyin method has good potential efficacy against AR. In this study, the pathogenesis of AR was found to be associated with *OPRD1* and *OPRM1*. However, the exact mechanism remains to be further investigated.

## 5. Conclusion

This study revealed that the Yiqi Qingre Ziyin method may improve the immune microenvironment of the nasal mucosa by affecting the expression of neuropeptide-related genes (including *DPP4*, *OPRD1*, and *OPRM1*), which provides a theoretical basis for further elucidating the molecular mechanisms of this method in the treatment of AR.

## Figures and Tables

**Table 1:** Virtual docking of five ingredients for DS targets.

Proteins		DPP4	OPRD1	OPRM1
Binding energy (kcal∗mol^−1^)	3-*beta*-Hydroxymethyllenetanshiquinone	-8.9	-8.3	-8.9
	11-Hydroxyrankinidine	—	—	-7.4
	7-Methoxy-2-methyl isoflavone	-7.8	—	-8.9
	Frutinone A	-8.6	—	—
	Luteolin	-7.6	—	—

## Data Availability

Genes related to atrophic rhinitis were gathered from 4 datasets, including GeneCards (https://www.genecards.org/), OMIM (https://omim.org/), PharmGKB (https://www.pharmgkb.org), and DrugBank (https://www.drugbank.com). The RCSB PDB database (http://www.rcsb.org/) and the ZINC database (http://zinc.docking.org/) were used to obtain the structures of target proteins and ligands.

## Abbreviations

AMK: Atractylodes macrocephala Koidz
AKT1: AKT serine/threonine kinase 1
AR: Atrophic rhinitis
BP: Biological processes
CASP3: Caspase 3
CC: Cellular components
CXCL8: C-X-C motif chemokine ligand 8
DS: Dangshen
DPP4: Dipeptidyl peptidase 4
GO: Gene Ontology
HMOX1: Heme oxygenase 1
HIF1A: Hypoxia-inducible factor 1-alpha
KEGG: Kyoto Encyclopedia of Genes and Genomes
LIA: Ligustrum lucidum Ait
MMP2: Matrix metallopeptidase 2
MMP9: Matrix metallopeptidase 9
MDS: Molecular dynamics simulation
MF: Molecular functions
NANC: Nonadrenergic noncholinergic nervous system
OPRD1: Opioid receptor delta 1
OPRM1: Opioid receptor mu 1
PPARG: Peroxisome proliferator-activated receptor gamma
PTGS2: Prostaglandin-endoperoxide synthase 2
MAPK14: Recombinant mitogen-activated protein kinase 14
MAPK8: Recombinant mitogen-activated protein kinase 8
RPR: Red peony root
TCMSP: Traditional Chinese Medicine Systematic Pharmacology Database Analysis Platform
TCM: Traditional Chinese medicines
TP53: Tumor protein P53
VCAM1: Vascular cell adhesion molecule 1.

## Supplementary Materials

Supplementary Materials Supplementary Table 1: enrichment analysis of GO suggests that the Yiqi Qingre Ziyin method presents a multitarget and multipathway action for the treatment of AR. Supplementary Table 2: in the KEGG metabolic pathway enrichment, a total of 78 relevant metabolic pathways were obtained at *P* < 0.05.

## References

[1] Bakshi S. S. (2019). Atrophic rhinitis. *The Journal of Allergy and Clinical Immunology. In Practice*.

[2] Shehata M. A. (1996). Atrophic rhinitis. *American Journal of Otolaryngology*.

[3] Mishra A., Kawatra R., Gola M., Cochrane ENT Group (2012). Interventions for atrophic rhinitis. *Cochrane Database of Systematic Reviews*.

[4] Hildenbrand T., Weber R. K., Brehmer D. (2011). Rhinitis sicca, dry nose and atrophic rhinitis: a review of the literature. *European Archives of Oto-Rhino-Laryngology*.

[5] Kang X., Chen B., Chen Y. S. (2020). A prediction modeling based on SNOT-22 score for endoscopic nasal septoplasty: a retrospective study. *Peer J*.

[6] Buto J. (1970). Atrophic rhinitis. *Jibiinkoka.*.

[7] McDonald J. L., Smith P. K., Smith C. A. (2016). Effect of acupuncture on house dust mite specific IgE, substance P, and symptoms in persistent allergic rhinitis. *Annals of Allergy, Asthma & Immunology*.

[8] Wang L., Chen M., Xu M. (2020). Effect of posterior nasal neurectomy on the suppression of allergic rhinitis. *American Journal of Otolaryngology*.

[9] Liang X., Wang Q., Jiang Z. (2020). Clinical research linking traditional Chinese medicine constitution types with diseases: a literature review of 1639 observational studies. *Journal of Traditional Chinese Medicine*.

[10] Gawlik R., DuBuske L. (2010). Mediator release of neuropeptides after nasal provocation in perennial allergic rhinitis patients. *Rhinology*.

[11] Kashiwabara M., Asano K., Mizuyoshi T., Kobayashi H. (2016). Suppression of neuropeptide production by quercetin in allergic rhinitis model rats. *BMC Complementary and Alternative Medicine*.

[12] McDonald J. L., Cripps A. W., Smith P. K., Smith C. A., Xue C. C., Golianu B. (2013). The anti-inflammatory effects of acupuncture and their relevance to allergic rhinitis: a narrative review and proposed model. *Evidence-Based Complementary and Alternative Medicine*.

[13] Sarin S., Undem B., Sanico A., Togias A. (2006). The role of the nervous system in rhinitis. *The Journal of Allergy and Clinical Immunology*.

[14] Zhang W., Chao X., Wu J. Q. (2021). Exploring the potential mechanism of Guchang Zhixie Wan for treating ulcerative colitis by comprehensive network pharmacological approaches and molecular docking validation as well as cell experiments. *Chemistry & Biodiversity*.

[15] Ru J., Li P., Wang J. (2014). TCMSP: a database of systems pharmacology for drug discovery from herbal medicines. *Journal of Cheminformatics*.

[16] Wang B., Liu Y., Sun J., Zhang N., Zheng X., Liu Q. (2021). Exploring the potential mechanism of Xiaokui Jiedu decoction for ulcerative colitis based on network pharmacology and molecular docking. *Journal of Healthcare Engineering*.

[17] Wang H., Zhang J., Zhu Q., Fu X., Li C. (2021). Integrating network pharmacology and experimental validation deciphers the mechanism of Guizhi Fuling Wan against adenomyosis. *Evidence-based Complementary and Alternative Medicine*.

[18] Hur B., Kang D., Lee S., Moon J. H., Lee G., Kim S. (2019). Venn-diaNet : venn diagram based network propagation analysis framework for comparing multiple biological experiments. *BMC Bioinformatics*.

[19] Shannon P., Markiel A., Ozier O. (2003). Cytoscape: a software environment for integrated models of biomolecular interaction networks. *Genome Research*.

[20] Szklarczyk D., Gable A. L., Lyon D. (2019). STRING v11: protein-protein association networks with increased coverage, supporting functional discovery in genome-wide experimental datasets. *Nucleic Acids Research*.

[21] Wu J. Y., Qin J., Li L. (2021). Roles of the immune/methylation/autophagy landscape on single-cell genotypes and stroke risk in breast cancer microenvironment. *Oxidative Medicine and Cellular Longevity*.

[22] Szklarczyk D., Morris J. H., Cook H. (2017). The STRING database in 2017: quality-controlled protein-protein association networks, made broadly accessible. *Nucleic Acids Research*.

[23] Yu G., Wang L.-G., Han Y., He Q.-Y. (2012). clusterProfiler: an R package for comparing biological themes among gene clusters. *Omics: A Journal of Integrative Biology*.

[24] Korthauer K., Kimes P. K., Duvallet C. (2019). A practical guide to methods controlling false discoveries in computational biology. *Genome Biology*.

[25] Chen Y., Chen C., Xiao X., Huang Z., Huang X., Yao W. (2021). TNF-*α* induces neutrophil apoptosis delay and promotes intestinal ischemia- reperfusion-induced lung injury through activating JNK/FoxO3a pathway. *Oxidative Medicine and Cellular Longevity*.

[26] Lin W., Wang Y., Chen Y., Wang Q., Gu Z., Zhu Y. (2021). Role of calcium signaling pathway-related gene regulatory networks in ischemic stroke based on multiple WGCNA and single-cell analysis. *Oxidative Medicine and Cellular Longevity*.

[27] Noureddine O., Gatfaoui S., Brandan S. A., Sagaama A., Marouani H., Issaoui N. (2020). Experimental and DFT studies on the molecular structure, spectroscopic properties, and molecular docking of 4-phenylpiperazine-1-ium dihydrogen phosphate. *Journal of Molecular Structure*.

[28] Burley S. K., Berman H. M., Bhikadiya C. (2019). RCSB Protein Data Bank: biological macromolecular structures enabling research and education in fundamental biology, biomedicine, biotechnology and energy. *Nucleic Acids Research*.

[29] Sterling T., Irwin J. J. (2015). ZINC 15 – ligand discovery for everyone. *Journal of Chemical Information and Modeling*.

[30] Sousa da Silva A. W., Vranken W. F. (2012). ACPYPE - AnteChamber PYthon Parser interfacE. *BMC Research Notes*.

[31] Bernardi A., Faller R., Reith D., Kirschner K. N. (2019). ACPYPE update for nonuniform 1-4 scale factors: conversion of the GLYCAM06 force field from AMBER to GROMACS. *SoftwareX*.

[32] Huang J., Rauscher S., Nawrocki G. (2017). CHARMM36m: an improved force field for folded and intrinsically disordered proteins. *Nature Methods*.

[33] Abraham M. J., Murtola T., Schulz R. (2015). GROMACS: high performance molecular simulations through multi-level parallelism from laptops to supercomputers. *SoftwareX*.

[34] Berendsen H. J., Hess B., Lindahl E., Van Der Spoel D., Mark A. E., Groenhof G. (2005). GROMACS: fast, flexible, and free. *Journal of Computational Chemistry*.

[35] Assenov Y., Ramírez F., Schelhorn S.-E., Lengauer T., Albrecht M. (2008). Computing topological parameters of biological networks. *Bioinformatics*.

[36] Moore E. J., Kern E. B. (2001). Atrophic rhinitis: a review of 242 cases. *American Journal of Rhinology*.

[37] Baek K.-S., Yi Y. S., Son Y. J. (2016). *In vitro* and *in vivo* anti-inflammatory activities of Korean red ginseng- derived components. *Journal of Ginseng Research*.

[38] Baek K.-S., Yi Y. S., Son Y. J. (2017). Comparison of anticancer activities of Korean red ginseng-derived fractions. *Journal of Ginseng Research*.

[39] Choi M. R., Kwak S. M., Bang S. H., Jeong J.-E., Kim D.-J. (2017). Chronic saponin treatment attenuates damage to the pancreas in chronic alcohol-treated diabetic rats. *Journal of Ginseng Research*.

[40] López-Lázaro M. (2009). Distribution and biological activities of the flavonoid luteolin. *Mini Reviews in Medicinal Chemistry*.

[41] Yu T., Yang Y., Kwak Y. S. (2017). Ginsenoside Rc from *Panax ginseng* exerts anti-inflammatory activity by targeting TANK-binding kinase 1/interferon regulatory factor-3 and p38/ATF-2. *Journal of Ginseng Research*.

[42] Yasui T., Ideno Y., Onizuka Y. (2019). The association of urinary estrogen levels with urinary isoflavone levels: difference between premenopausal women and postmenopausal women. *Maturitas*.

[43] Amato P., Young R. L., Steinberg F. M. (2013). Effect of soy isoflavone supplementation on menopausal quality of life. *Menopause*.

[44] Cui Y., Huang C., Momma H., Niu K., Nagatomi R. (2020). Daily dietary isoflavone intake in relation to lowered risk of depressive symptoms among men. *Journal of Affective Disorders*.

[45] Gorrell M. D., Gysbers V., McCaughan G. W. (2001). CD26: a multifunctional integral membrane and secreted protein of activated lymphocytes. *Scandinavian Journal of Immunology*.

[46] Bengsch B., Seigel B., Flecken T., Wolanski J., Blum H. E., Thimme R. (2012). Human Th17 cells express high levels of enzymatically active dipeptidylpeptidase IV (CD26). *The Journal of Immunology*.

[47] Ellingsen T., Hornung N., Møller B. K., Hjelm-Poulsen J., Stengaard-Pedersen K. (2007). In active chronic rheumatoid arthritis, dipeptidyl peptidase IV density is increased on monocytes and CD4+ T lymphocytes. *Scandinavian Journal of Immunology*.

[48] Krakauer M., Sorensen P. S., Sellebjerg F. (2006). CD4+ memory T cells with high CD26 surface expression are enriched for Th1 markers and correlate with clinical severity of multiple sclerosis. *Journal of Neuroimmunology*.

[49] Jensen J., Langkilde A. R., Fenst C. (2004). CD4 T cell activation and disease activity at onset of multiple sclerosis. *Journal of Neuroimmunology*.

[50] Jensen J., Langkilde A. R., Frederiksen J. L., Sellebjerg F. (2006). CD8^+^ T cell activation correlates with disease activity in clinically isolated syndromes and is regulated by interferon-*β* treatment. *Journal of Neuroimmunology*.

[51] Crist R. C., Clarke T.-K. (2018). OPRD1 genetic variation and human disease. *Handbook of Experimental Pharmacology*.

[52] Grice D. E., Halmi K. A., Fichter M. M. (2002). Evidence for a susceptibility gene for anorexia nervosa on chromosome 1. *American Journal of Human Genetics*.

[53] Kaye W. H., Lilenfeld L. R., Berrettini W. H. (2000). A search for susceptibility loci for anorexia nervosa: methods and sample description. *Biological Psychiatry*.

[54] Vanderah T. W. (2010). Delta and kappa opioid receptors as suitable drug targets for pain. *The Clinical Journal of Pain*.

[55] Cahill C. M., Holdridge S. V., Morinville A. (2007). Trafficking of *δ*-opioid receptors and other G-protein-coupled receptors: implications for pain and analgesia. *Trends in Pharmacological Sciences*.

[56] Cahill C. M., Morinville A., Hoffert C., O’Donnell D., Beaudet A. (2003). Up-regulation and trafficking of *δ* opioid receptor in a model of chronic inflammation: implications for pain control. *Pain*.

