# Supplementary material for: Exploring the Mechanism of Yiqi Qingre Ziyin Method in Regulating Neuropeptide Expression for the Treatment of Atrophic Rhinitis
Source: Dis Markers. 2022 Mar 8;2022:4416637. doi: 10.1155/2022/4416637 (PMC8923799; doi:10.1155/2022/4416637)
Supplement: Supplementary Materials — Supplementary Table 1: enrichment analysis of GO suggests that the Yiqi Qingre Ziyin method presents a multitarget and multipathway action for the treatment of AR. Supplementary Table 2: in the KEGG metabolic pathway enrichment, a total of 78 relevant metabolic pathways were obtained at P < 0.05. [file 4416637.f1.docx]

| Supplementary table 1. Enrichment analysis of GO | | | | | | | | | |
| --- | --- | --- | --- | --- | --- | --- | --- | --- | --- |
| ONTOLOGY | ID | Description | GeneRatio | BgRatio | pvalue | p.adjust | qvalue | geneID | Count |
| BP | GO:0002526 | acute inflammatory response | 5/39 | 108/18670 | 2.90E-06 | 5.92E-05 | 2.61E-05 | PTGS2/IL6ST/PPARG/ICAM1/VCAM1 | 5 |
| BP | GO:0007568 | aging | 11/39 | 321/18670 | 3.57E-11 | 5.87E-09 | 2.58E-09 | PTGS2/RELA/AKT1/BCL2/TP53/MPO/JUN/MAPK14/CYP1A1/ICAM1/VCAM1 | 11 |
| BP | GO:0001667 | ameboidal-type cell migration | 9/39 | 462/18670 | 3.53E-07 | 9.99E-06 | 4.40E-06 | PTGS2/MMP9/AKT1/HIF1A/JUN/PRKCA/PPARG/KDR/HMOX1 | 9 |
| BP | GO:0008637 | apoptotic mitochondrial changes | 6/39 | 124/18670 | 2.07E-07 | 6.46E-06 | 2.85E-06 | MMP9/AKT1/BCL2/TP53/JUN/MAPK8 | 6 |
| BP | GO:0006914 | autophagy | 8/39 | 496/18670 | 6.99E-06 | 0.000117139 | 5.16E-05 | AKT1/BCL2/CASP3/TP53/HIF1A/KDR/MAPK8/HMOX1 | 8 |
| BP | GO:0001782 | B cell homeostasis | 3/39 | 30/18670 | 3.29E-05 | 0.000401711 | 0.000176932 | BCL2/CASP3/HIF1A | 3 |
| BP | GO:0043534 | blood vessel endothelial cell migration | 7/39 | 180/18670 | 8.17E-08 | 3.05E-06 | 1.34E-06 | PTGS2/AKT1/HIF1A/PRKCA/PPARG/KDR/HMOX1 | 7 |
| BP | GO:0060444 | branching involved in mammary gland duct morphogenesis | 3/39 | 25/18670 | 1.88E-05 | 0.000262858 | 0.000115775 | PGR/AR/ESR1 | 3 |
| BP | GO:0007569 | cell aging | 4/39 | 116/18670 | 9.84E-05 | 0.000950609 | 0.000418693 | BCL2/TP53/MAPK14/ICAM1 | 4 |
| BP | GO:0002042 | cell migration involved in sprouting angiogenesis | 4/39 | 98/18670 | 5.10E-05 | 0.000560866 | 0.000247032 | PTGS2/AKT1/KDR/HMOX1 | 4 |
| BP | GO:1990748 | cellular detoxification | 4/39 | 112/18670 | 8.58E-05 | 0.00085293 | 0.000375671 | PTGS2/GSTM1/PTGS1/MPO | 4 |
| BP | GO:0071214 | cellular response to abiotic stimulus | 6/39 | 331/18670 | 5.91E-05 | 0.000627808 | 0.000276517 | PTGS2/AKT1/CASP3/TP53/MAPK14/MAPK8 | 6 |
| BP | GO:0071216 | cellular response to biotic stimulus | 10/39 | 236/18670 | 3.97E-11 | 6.11E-09 | 2.69E-09 | RELA/NFKBIA/CXCL8/AKT1/TP53/PRKCA/NOS2/MAPK14/MAPK8/ICAM1 | 10 |
| BP | GO:0071276 | cellular response to cadmium ion | 6/39 | 37/18670 | 1.23E-10 | 1.69E-08 | 7.42E-09 | MMP9/AKT1/JUN/NCF1/MAPK8/HMOX1 | 6 |
| BP | GO:0062197 | cellular response to chemical stress | 15/39 | 350/18670 | 1.54E-16 | 1.27E-13 | 5.57E-14 | PTGS2/RELA/MMP2/MMP9/AKT1/BCL2/CASP3/TP53/HIF1A/MPO/JUN/PPARG/NCF1/MAPK8/HMOX1 | 15 |
| BP | GO:0036294 | cellular response to decreased oxygen levels | 7/39 | 217/18670 | 2.92E-07 | 8.46E-06 | 3.72E-06 | PTGS2/AKT1/BCL2/TP53/HIF1A/HMOX1/ICAM1 | 7 |
| BP | GO:0035690 | cellular response to drug | 8/39 | 433/18670 | 2.57E-06 | 5.36E-05 | 2.36E-05 | PTGS2/RELA/TP53/AHR/NOS2/KDR/HMOX1/ICAM1 | 8 |
| BP | GO:0104004 | cellular response to environmental stimulus | 6/39 | 331/18670 | 5.91E-05 | 0.000627808 | 0.000276517 | PTGS2/AKT1/CASP3/TP53/MAPK14/MAPK8 | 6 |
| BP | GO:0071496 | cellular response to external stimulus | 10/39 | 339/18670 | 1.36E-09 | 1.20E-07 | 5.27E-08 | PTGS2/AKT1/BCL2/TP53/JUN/PPARG/MAPK8/HMOX1/ICAM1/VCAM1 | 10 |
| BP | GO:0031668 | cellular response to extracellular stimulus | 9/39 | 268/18670 | 3.29E-09 | 2.61E-07 | 1.15E-07 | PTGS2/BCL2/TP53/JUN/PPARG/MAPK8/HMOX1/ICAM1/VCAM1 | 9 |
| BP | GO:0071456 | cellular response to hypoxia | 7/39 | 207/18670 | 2.12E-07 | 6.46E-06 | 2.85E-06 | PTGS2/AKT1/BCL2/TP53/HIF1A/HMOX1/ICAM1 | 7 |
| BP | GO:0071241 | cellular response to inorganic substance | 8/39 | 215/18670 | 1.23E-08 | 7.57E-07 | 3.33E-07 | PTGS2/MMP9/AKT1/JUN/NCF1/MAPK8/HMOX1/CYP1A1 | 8 |
| BP | GO:0071346 | cellular response to interferon-gamma | 5/39 | 180/18670 | 3.48E-05 | 0.00042007 | 0.000185019 | TP53/NOS2/PPARG/ICAM1/VCAM1 | 5 |
| BP | GO:0071347 | cellular response to interleukin-1 | 6/39 | 179/18670 | 1.79E-06 | 4.10E-05 | 1.81E-05 | RELA/NFKBIA/CXCL8/HIF1A/IKBKB/ICAM1 | 6 |
| BP | GO:1901655 | cellular response to ketone | 5/39 | 93/18670 | 1.39E-06 | 3.34E-05 | 1.47E-05 | AR/AKT1/AHR/PPARG/ICAM1 | 5 |
| BP | GO:0071222 | cellular response to lipopolysaccharide | 9/39 | 205/18670 | 3.10E-10 | 4.02E-08 | 1.77E-08 | RELA/NFKBIA/CXCL8/AKT1/PRKCA/NOS2/MAPK14/MAPK8/ICAM1 | 9 |
| BP | GO:0071248 | cellular response to metal ion | 8/39 | 188/18670 | 4.29E-09 | 3.09E-07 | 1.36E-07 | PTGS2/MMP9/AKT1/JUN/NCF1/MAPK8/HMOX1/CYP1A1 | 8 |
| BP | GO:0071219 | cellular response to molecule of bacterial origin | 9/39 | 212/18670 | 4.18E-10 | 4.88E-08 | 2.15E-08 | RELA/NFKBIA/CXCL8/AKT1/PRKCA/NOS2/MAPK14/MAPK8/ICAM1 | 9 |
| BP | GO:0031669 | cellular response to nutrient levels | 8/39 | 237/18670 | 2.63E-08 | 1.41E-06 | 6.19E-07 | PTGS2/BCL2/TP53/JUN/PPARG/MAPK8/HMOX1/ICAM1 | 8 |
| BP | GO:0034599 | cellular response to oxidative stress | 12/39 | 302/18670 | 6.83E-13 | 1.68E-10 | 7.42E-11 | RELA/MMP2/MMP9/AKT1/BCL2/TP53/HIF1A/MPO/JUN/NCF1/MAPK8/HMOX1 | 12 |
| BP | GO:0071453 | cellular response to oxygen levels | 8/39 | 234/18670 | 2.38E-08 | 1.30E-06 | 5.74E-07 | PTGS2/AKT1/BCL2/TP53/HIF1A/PPARG/HMOX1/ICAM1 | 8 |
| BP | GO:0034614 | cellular response to reactive oxygen species | 8/39 | 168/18670 | 1.76E-09 | 1.50E-07 | 6.60E-08 | RELA/MMP2/MMP9/AKT1/MPO/JUN/NCF1/MAPK8 | 8 |
| BP | GO:0071383 | cellular response to steroid hormone stimulus | 6/39 | 248/18670 | 1.17E-05 | 0.000176634 | 7.78E-05 | PGR/AR/ESR1/PPARG/ESR2/ICAM1 | 6 |
| BP | GO:0097237 | cellular response to toxic substance | 7/39 | 247/18670 | 6.98E-07 | 1.81E-05 | 7.98E-06 | PTGS2/RELA/GSTM1/PTGS1/MPO/KDR/HMOX1 | 7 |
| BP | GO:0071356 | cellular response to tumor necrosis factor | 8/39 | 291/18670 | 1.28E-07 | 4.38E-06 | 1.93E-06 | RELA/NFKBIA/CXCL8/AKT1/MAPK14/IKBKB/ICAM1/VCAM1 | 8 |
| BP | GO:0035924 | cellular response to vascular endothelial growth factor stimulus | 5/39 | 68/18670 | 2.89E-07 | 8.46E-06 | 3.72E-06 | RELA/AKT1/MAPK14/KDR/VCAM1 | 5 |
| BP | GO:0071466 | cellular response to xenobiotic stimulus | 5/39 | 180/18670 | 3.48E-05 | 0.00042007 | 0.000185019 | GSTM1/PTGS1/AHR/CYP1A1/ICAM1 | 5 |
| BP | GO:0007623 | circadian rhythm | 6/39 | 208/18670 | 4.27E-06 | 8.00E-05 | 3.52E-05 | TP53/AHR/JUN/NOS2/PPARG/MAPK8 | 6 |
| BP | GO:0032963 | collagen metabolic process | 5/39 | 115/18670 | 3.96E-06 | 7.62E-05 | 3.36E-05 | MMP2/MMP9/HIF1A/PPARG/MMP1 | 5 |
| BP | GO:0046545 | development of primary female sexual characteristics | 5/39 | 101/18670 | 2.09E-06 | 4.59E-05 | 2.02E-05 | PGR/ESR1/BCL2/CASP3/ICAM1 | 5 |
| BP | GO:0045137 | development of primary sexual characteristics | 6/39 | 223/18670 | 6.37E-06 | 0.00011135 | 4.90E-05 | PGR/AR/ESR1/BCL2/CASP3/ICAM1 | 6 |
| BP | GO:0006352 | DNA-templated transcription, initiation | 7/39 | 249/18670 | 7.37E-07 | 1.89E-05 | 8.34E-06 | PGR/AR/ESR1/TP53/JUN/PPARG/ESR2 | 7 |
| BP | GO:0043542 | endothelial cell migration | 7/39 | 274/18670 | 1.40E-06 | 3.34E-05 | 1.47E-05 | PTGS2/AKT1/HIF1A/PRKCA/PPARG/KDR/HMOX1 | 7 |
| BP | GO:0001935 | endothelial cell proliferation | 7/39 | 191/18670 | 1.23E-07 | 4.25E-06 | 1.87E-06 | AKT1/HIF1A/JUN/PRKCA/PPARG/KDR/HMOX1 | 7 |
| BP | GO:0002064 | epithelial cell development | 6/39 | 207/18670 | 4.15E-06 | 7.87E-05 | 3.47E-05 | PGR/AR/ESR1/HIF1A/IKBKB/ICAM1 | 6 |
| BP | GO:0010631 | epithelial cell migration | 9/39 | 352/18670 | 3.50E-08 | 1.72E-06 | 7.59E-07 | PTGS2/MMP9/AKT1/HIF1A/JUN/PRKCA/PPARG/KDR/HMOX1 | 9 |
| BP | GO:0050673 | epithelial cell proliferation | 10/39 | 434/18670 | 1.45E-08 | 8.28E-07 | 3.65E-07 | PGR/AR/ESR1/AKT1/HIF1A/JUN/PRKCA/PPARG/KDR/HMOX1 | 10 |
| BP | GO:0060562 | epithelial tube morphogenesis | 6/39 | 322/18670 | 5.07E-05 | 0.000560498 | 0.00024687 | PGR/AR/ESR1/BCL2/CASP3/HIF1A | 6 |
| BP | GO:0090132 | epithelium migration | 9/39 | 355/18670 | 3.76E-08 | 1.82E-06 | 8.01E-07 | PTGS2/MMP9/AKT1/HIF1A/JUN/PRKCA/PPARG/KDR/HMOX1 | 9 |
| BP | GO:0097191 | extrinsic apoptotic signaling pathway | 7/39 | 224/18670 | 3.62E-07 | 1.01E-05 | 4.46E-06 | AR/RELA/AKT1/BCL2/CASP3/HMOX1/ICAM1 | 7 |
| BP | GO:1901568 | fatty acid derivative metabolic process | 5/39 | 167/18670 | 2.43E-05 | 0.000321607 | 0.000141651 | PTGS2/PTGS1/PON1/CYP1A1/ALOX5 | 5 |
| BP | GO:0006631 | fatty acid metabolic process | 8/39 | 383/18670 | 1.03E-06 | 2.53E-05 | 1.12E-05 | PTGS2/PTGS1/AKT1/PON1/PPARG/MAPK14/CYP1A1/ALOX5 | 8 |
| BP | GO:0008585 | female gonad development | 5/39 | 96/18670 | 1.62E-06 | 3.77E-05 | 1.66E-05 | PGR/ESR1/BCL2/CASP3/ICAM1 | 5 |
| BP | GO:0007565 | female pregnancy | 8/39 | 192/18670 | 5.06E-09 | 3.47E-07 | 1.53E-07 | PTGS2/PGR/AR/ESR1/MMP2/MMP9/AKT1/BCL2 | 8 |
| BP | GO:0046660 | female sex differentiation | 5/39 | 115/18670 | 3.96E-06 | 7.62E-05 | 3.36E-05 | PGR/ESR1/BCL2/CASP3/ICAM1 | 5 |
| BP | GO:0048144 | fibroblast proliferation | 4/39 | 84/18670 | 2.78E-05 | 0.000358798 | 0.000158032 | ESR1/TP53/JUN/PPARG | 4 |
| BP | GO:0048732 | gland development | 10/39 | 434/18670 | 1.45E-08 | 8.28E-07 | 3.65E-07 | PGR/AR/ESR1/RELA/AKT1/BCL2/HIF1A/JUN/HMOX1/CYP1A1 | 10 |
| BP | GO:0034349 | glial cell apoptotic process | 3/39 | 15/18670 | 3.77E-06 | 7.47E-05 | 3.29E-05 | CASP3/TP53/PRKCA | 3 |
| BP | GO:0008406 | gonad development | 6/39 | 217/18670 | 5.45E-06 | 9.80E-05 | 4.32E-05 | PGR/AR/ESR1/BCL2/CASP3/ICAM1 | 6 |
| BP | GO:0048872 | homeostasis of number of cells | 6/39 | 246/18670 | 1.12E-05 | 0.000171849 | 7.57E-05 | AKT1/BCL2/CASP3/HIF1A/MAPK14/HMOX1 | 6 |
| BP | GO:0006690 | icosanoid metabolic process | 5/39 | 114/18670 | 3.79E-06 | 7.47E-05 | 3.29E-05 | PTGS2/PTGS1/PON1/CYP1A1/ALOX5 | 5 |
| BP | GO:0007249 | I-kappaB kinase/NF-kappaB signaling | 6/39 | 269/18670 | 1.85E-05 | 0.000260752 | 0.000114848 | ESR1/RELA/NFKBIA/AKT1/IKBKB/HMOX1 | 6 |
| BP | GO:0051170 | import into nucleus | 5/39 | 163/18670 | 2.16E-05 | 0.000290892 | 0.000128123 | PTGS2/NFKBIA/AKT1/TP53/MAPK14 | 5 |
| BP | GO:0060333 | interferon-gamma-mediated signaling pathway | 4/39 | 91/18670 | 3.81E-05 | 0.00045161 | 0.000198911 | TP53/PPARG/ICAM1/VCAM1 | 4 |
| BP | GO:0030522 | intracellular receptor signaling pathway | 8/39 | 278/18670 | 9.01E-08 | 3.31E-06 | 1.46E-06 | PGR/AR/ESR1/RELA/NFKBIA/AHR/PPARG/ESR2 | 8 |
| BP | GO:0097193 | intrinsic apoptotic signaling pathway | 8/39 | 289/18670 | 1.21E-07 | 4.25E-06 | 1.87E-06 | PTGS2/MMP9/AKT1/BCL2/CASP3/TP53/HIF1A/HMOX1 | 8 |
| BP | GO:0008631 | intrinsic apoptotic signaling pathway in response to oxidative stress | 3/39 | 43/18670 | 9.82E-05 | 0.000950609 | 0.000418693 | AKT1/BCL2/HIF1A | 3 |
| BP | GO:0061756 | leukocyte adhesion to vascular endothelial cell | 4/39 | 44/18670 | 2.08E-06 | 4.59E-05 | 2.02E-05 | RELA/ICAM1/SELE/VCAM1 | 4 |
| BP | GO:0071887 | leukocyte apoptotic process | 4/39 | 104/18670 | 6.43E-05 | 0.000665925 | 0.000293305 | AKT1/CASP3/TP53/HIF1A | 4 |
| BP | GO:0007159 | leukocyte cell-cell adhesion | 8/39 | 337/18670 | 3.92E-07 | 1.07E-05 | 4.72E-06 | RELA/IL6ST/AKT1/CASP3/IL4R/ICAM1/SELE/VCAM1 | 8 |
| BP | GO:0001776 | leukocyte homeostasis | 4/39 | 86/18670 | 3.05E-05 | 0.000383657 | 0.000168981 | AKT1/BCL2/CASP3/HIF1A | 4 |
| BP | GO:0050900 | leukocyte migration | 8/39 | 499/18670 | 7.30E-06 | 0.000121592 | 5.36E-05 | CXCL8/AKT1/MAPK14/MMP1/HMOX1/ICAM1/SELE/VCAM1 | 8 |
| BP | GO:0070661 | leukocyte proliferation | 6/39 | 298/18670 | 3.29E-05 | 0.000401711 | 0.000176932 | IL6ST/BCL2/CASP3/TP53/AHR/VCAM1 | 6 |
| BP | GO:0031663 | lipopolysaccharide-mediated signaling pathway | 4/39 | 58/18670 | 6.36E-06 | 0.00011135 | 4.90E-05 | NFKBIA/AKT1/PRKCA/MAPK14 | 4 |
| BP | GO:0019372 | lipoxygenase pathway | 3/39 | 16/18670 | 4.63E-06 | 8.45E-05 | 3.72E-05 | PTGS2/PON1/ALOX5 | 3 |
| BP | GO:0042759 | long-chain fatty acid biosynthetic process | 3/39 | 30/18670 | 3.29E-05 | 0.000401711 | 0.000176932 | PTGS2/CYP1A1/ALOX5 | 3 |
| BP | GO:0001676 | long-chain fatty acid metabolic process | 4/39 | 109/18670 | 7.72E-05 | 0.000776838 | 0.000342157 | PTGS2/PTGS1/CYP1A1/ALOX5 | 4 |
| BP | GO:0002260 | lymphocyte homeostasis | 4/39 | 62/18670 | 8.31E-06 | 0.000133839 | 5.89E-05 | AKT1/BCL2/CASP3/HIF1A | 4 |
| BP | GO:0046651 | lymphocyte proliferation | 6/39 | 272/18670 | 1.97E-05 | 0.000271394 | 0.000119535 | IL6ST/BCL2/CASP3/TP53/AHR/VCAM1 | 6 |
| BP | GO:0016236 | macroautophagy | 7/39 | 295/18670 | 2.28E-06 | 4.89E-05 | 2.16E-05 | AKT1/CASP3/TP53/HIF1A/KDR/MAPK8/HMOX1 | 7 |
| BP | GO:0060749 | mammary gland alveolus development | 3/39 | 18/18670 | 6.73E-06 | 0.000115133 | 5.07E-05 | AR/ESR1/HIF1A | 3 |
| BP | GO:0030879 | mammary gland development | 5/39 | 143/18670 | 1.15E-05 | 0.000174504 | 7.69E-05 | PGR/AR/ESR1/AKT1/HIF1A | 5 |
| BP | GO:0060603 | mammary gland duct morphogenesis | 3/39 | 33/18670 | 4.40E-05 | 0.000499917 | 0.000220188 | PGR/AR/ESR1 | 3 |
| BP | GO:0061180 | mammary gland epithelium development | 5/39 | 72/18670 | 3.85E-07 | 1.07E-05 | 4.70E-06 | PGR/AR/ESR1/AKT1/HIF1A | 5 |
| BP | GO:0061377 | mammary gland lobule development | 3/39 | 18/18670 | 6.73E-06 | 0.000115133 | 5.07E-05 | AR/ESR1/HIF1A | 3 |
| BP | GO:0060135 | maternal process involved in female pregnancy | 5/39 | 64/18670 | 2.12E-07 | 6.46E-06 | 2.85E-06 | PTGS2/PGR/AR/ESR1/AKT1 | 5 |
| BP | GO:0032943 | mononuclear cell proliferation | 6/39 | 274/18670 | 2.05E-05 | 0.000281272 | 0.000123885 | IL6ST/BCL2/CASP3/TP53/AHR/VCAM1 | 6 |
| BP | GO:0044706 | multi-multicellular organism process | 9/39 | 222/18670 | 6.28E-10 | 6.73E-08 | 2.96E-08 | PTGS2/PGR/AR/ESR1/MMP2/MMP9/AKT1/BCL2/CYP1A1 | 9 |
| BP | GO:0033002 | muscle cell proliferation | 8/39 | 239/18670 | 2.80E-08 | 1.44E-06 | 6.34E-07 | PTGS2/MMP2/MMP9/AKT1/JUN/PPARG/MAPK14/HMOX1 | 8 |
| BP | GO:0030099 | myeloid cell differentiation | 8/39 | 416/18670 | 1.91E-06 | 4.31E-05 | 1.90E-05 | MMP9/NFKBIA/CASP3/HIF1A/JUN/PRKCA/PPARG/MAPK14 | 8 |
| BP | GO:0002573 | myeloid leukocyte differentiation | 5/39 | 204/18670 | 6.31E-05 | 0.000656205 | 0.000289024 | MMP9/JUN/PRKCA/PPARG/MAPK14 | 5 |
| BP | GO:2001234 | negative regulation of apoptotic signaling pathway | 9/39 | 230/18670 | 8.59E-10 | 8.35E-08 | 3.68E-08 | PTGS2/AR/RELA/MMP9/AKT1/BCL2/HIF1A/HMOX1/ICAM1 | 9 |
| BP | GO:0010507 | negative regulation of autophagy | 4/39 | 84/18670 | 2.78E-05 | 0.000358798 | 0.000158032 | AKT1/BCL2/TP53/HMOX1 | 4 |
| BP | GO:0051100 | negative regulation of binding | 5/39 | 169/18670 | 2.57E-05 | 0.000336919 | 0.000148395 | NFKBIA/AKT1/JUN/MAPK8/HMOX1 | 5 |
| BP | GO:0009895 | negative regulation of catabolic process | 7/39 | 308/18670 | 3.04E-06 | 6.14E-05 | 2.70E-05 | RELA/AKT1/BCL2/TP53/NOS2/MAPK14/HMOX1 | 7 |
| BP | GO:2001237 | negative regulation of extrinsic apoptotic signaling pathway | 6/39 | 104/18670 | 7.26E-08 | 2.79E-06 | 1.23E-06 | AR/RELA/AKT1/BCL2/HMOX1/ICAM1 | 6 |
| BP | GO:0060969 | negative regulation of gene silencing | 3/39 | 34/18670 | 4.82E-05 | 0.000535174 | 0.000235716 | ESR1/TP53/PPARG | 3 |
| BP | GO:0060965 | negative regulation of gene silencing by miRNA | 3/39 | 16/18670 | 4.63E-06 | 8.45E-05 | 3.72E-05 | ESR1/TP53/PPARG | 3 |
| BP | GO:0060967 | negative regulation of gene silencing by RNA | 3/39 | 19/18670 | 7.98E-06 | 0.000129346 | 5.70E-05 | ESR1/TP53/PPARG | 3 |
| BP | GO:2001243 | negative regulation of intrinsic apoptotic signaling pathway | 5/39 | 98/18670 | 1.80E-06 | 4.10E-05 | 1.81E-05 | PTGS2/MMP9/AKT1/BCL2/HIF1A | 5 |
| BP | GO:0043271 | negative regulation of ion transport | 5/39 | 157/18670 | 1.80E-05 | 0.000255338 | 0.000112463 | PTGS2/MMP9/AKT1/BCL2/ICAM1 | 5 |
| BP | GO:0016242 | negative regulation of macroautophagy | 3/39 | 33/18670 | 4.40E-05 | 0.000499917 | 0.000220188 | AKT1/TP53/HMOX1 | 3 |
| BP | GO:1901215 | negative regulation of neuron death | 5/39 | 208/18670 | 6.92E-05 | 0.000702535 | 0.00030943 | AKT1/BCL2/HIF1A/JUN/HMOX1 | 5 |
| BP | GO:0060149 | negative regulation of posttranscriptional gene silencing | 3/39 | 19/18670 | 7.98E-06 | 0.000129346 | 5.70E-05 | ESR1/TP53/PPARG | 3 |
| BP | GO:0051402 | neuron apoptotic process | 6/39 | 239/18670 | 9.47E-06 | 0.00014954 | 6.59E-05 | BCL2/CASP3/TP53/HIF1A/JUN/HMOX1 | 6 |
| BP | GO:0070997 | neuron death | 7/39 | 348/18670 | 6.78E-06 | 0.000115133 | 5.07E-05 | AKT1/BCL2/CASP3/TP53/HIF1A/JUN/HMOX1 | 7 |
| BP | GO:0042136 | neurotransmitter biosynthetic process | 4/39 | 106/18670 | 6.93E-05 | 0.000702535 | 0.00030943 | PTGS2/AKT1/NOS2/ICAM1 | 4 |
| BP | GO:0006809 | nitric oxide biosynthetic process | 4/39 | 77/18670 | 1.97E-05 | 0.000271394 | 0.000119535 | PTGS2/AKT1/NOS2/ICAM1 | 4 |
| BP | GO:0046209 | nitric oxide metabolic process | 4/39 | 82/18670 | 2.53E-05 | 0.000333198 | 0.000146756 | PTGS2/AKT1/NOS2/ICAM1 | 4 |
| BP | GO:0035265 | organ growth | 5/39 | 204/18670 | 6.31E-05 | 0.000656205 | 0.000289024 | AR/ESR1/AKT1/BCL2/MAPK14 | 5 |
| BP | GO:0001503 | ossification | 7/39 | 398/18670 | 1.62E-05 | 0.000230734 | 0.000101626 | PTGS2/MMP2/IL6ST/AKT1/BCL2/HIF1A/MAPK14 | 7 |
| BP | GO:0030728 | ovulation | 3/39 | 21/18670 | 1.09E-05 | 0.000169228 | 7.45E-05 | PTGS2/PGR/IL4R | 3 |
| BP | GO:0018209 | peptidyl-serine modification | 7/39 | 322/18670 | 4.07E-06 | 7.78E-05 | 3.43E-05 | PTGS2/AKT1/BCL2/PRKCA/MAPK14/IKBKB/MAPK8 | 7 |
| BP | GO:0018105 | peptidyl-serine phosphorylation | 7/39 | 299/18670 | 2.50E-06 | 5.26E-05 | 2.32E-05 | PTGS2/AKT1/BCL2/PRKCA/MAPK14/IKBKB/MAPK8 | 7 |
| BP | GO:0001890 | placenta development | 5/39 | 152/18670 | 1.54E-05 | 0.000222208 | 9.79E-05 | PTGS2/AKT1/HIF1A/PPARG/MAPK14 | 5 |
| BP | GO:0045766 | positive regulation of angiogenesis | 6/39 | 204/18670 | 3.82E-06 | 7.47E-05 | 3.29E-05 | PTGS2/CXCL8/HIF1A/PRKCA/KDR/HMOX1 | 6 |
| BP | GO:0043536 | positive regulation of blood vessel endothelial cell migration | 6/39 | 79/18670 | 1.38E-08 | 8.28E-07 | 3.65E-07 | PTGS2/AKT1/HIF1A/PRKCA/KDR/HMOX1 | 6 |
| BP | GO:0045785 | positive regulation of cell adhesion | 8/39 | 403/18670 | 1.51E-06 | 3.57E-05 | 1.57E-05 | RELA/IL6ST/AKT1/PRKCA/IL4R/KDR/ICAM1/VCAM1 | 8 |
| BP | GO:0090050 | positive regulation of cell migration involved in sprouting angiogenesis | 3/39 | 37/18670 | 6.23E-05 | 0.000653622 | 0.000287886 | PTGS2/KDR/HMOX1 | 3 |
| BP | GO:0022409 | positive regulation of cell-cell adhesion | 6/39 | 255/18670 | 1.37E-05 | 0.000200719 | 8.84E-05 | RELA/IL6ST/AKT1/IL4R/ICAM1/VCAM1 | 6 |
| BP | GO:1903829 | positive regulation of cellular protein localization | 6/39 | 324/18670 | 5.25E-05 | 0.000571626 | 0.000251772 | PTGS2/AKT1/BCL2/TP53/MAPK14/MAPK8 | 6 |
| BP | GO:0031281 | positive regulation of cyclase activity | 3/39 | 22/18670 | 1.26E-05 | 0.000187217 | 8.25E-05 | NOS2/MAPK14/MAPK8 | 3 |
| BP | GO:0001819 | positive regulation of cytokine production | 7/39 | 464/18670 | 4.32E-05 | 0.000497788 | 0.00021925 | PTGS2/RELA/IL6ST/HIF1A/MAPK14/IL4R/HMOX1 | 7 |
| BP | GO:0051091 | positive regulation of DNA-binding transcription factor activity | 8/39 | 261/18670 | 5.54E-08 | 2.36E-06 | 1.04E-06 | AR/ESR1/RELA/AKT1/PPARG/ESR2/IKBKB/ICAM1 | 8 |
| BP | GO:2000144 | positive regulation of DNA-templated transcription, initiation | 3/39 | 27/18670 | 2.38E-05 | 0.000317106 | 0.000139669 | ESR1/TP53/JUN | 3 |
| BP | GO:0010595 | positive regulation of endothelial cell migration | 6/39 | 128/18670 | 2.50E-07 | 7.43E-06 | 3.27E-06 | PTGS2/AKT1/HIF1A/PRKCA/KDR/HMOX1 | 6 |
| BP | GO:0001938 | positive regulation of endothelial cell proliferation | 6/39 | 112/18670 | 1.13E-07 | 4.04E-06 | 1.78E-06 | AKT1/HIF1A/JUN/PRKCA/KDR/HMOX1 | 6 |
| BP | GO:0045742 | positive regulation of epidermal growth factor receptor signaling pathway | 3/39 | 31/18670 | 3.64E-05 | 0.000437235 | 0.000192579 | MMP9/AKT1/NCF1 | 3 |
| BP | GO:0010634 | positive regulation of epithelial cell migration | 8/39 | 171/18670 | 2.03E-09 | 1.67E-07 | 7.34E-08 | PTGS2/MMP9/AKT1/HIF1A/JUN/PRKCA/KDR/HMOX1 | 8 |
| BP | GO:0050679 | positive regulation of epithelial cell proliferation | 7/39 | 206/18670 | 2.05E-07 | 6.46E-06 | 2.85E-06 | AR/AKT1/HIF1A/JUN/PRKCA/KDR/HMOX1 | 7 |
| BP | GO:1901186 | positive regulation of ERBB signaling pathway | 3/39 | 33/18670 | 4.40E-05 | 0.000499917 | 0.000220188 | MMP9/AKT1/NCF1 | 3 |
| BP | GO:1904951 | positive regulation of establishment of protein localization | 7/39 | 456/18670 | 3.87E-05 | 0.000456415 | 0.000201027 | PTGS2/BCL2/TP53/HIF1A/MAPK14/IL4R/MAPK8 | 7 |
| BP | GO:0045600 | positive regulation of fat cell differentiation | 4/39 | 64/18670 | 9.44E-06 | 0.00014954 | 6.59E-05 | PTGS2/AKT1/PPARG/MAPK14 | 4 |
| BP | GO:0045687 | positive regulation of glial cell differentiation | 3/39 | 43/18670 | 9.82E-05 | 0.000950609 | 0.000418693 | RELA/IL6ST/PPARG | 3 |
| BP | GO:1903708 | positive regulation of hemopoiesis | 5/39 | 185/18670 | 3.96E-05 | 0.000465149 | 0.000204874 | HIF1A/JUN/PRKCA/MAPK14/IL4R | 5 |
| BP | GO:0090316 | positive regulation of intracellular protein transport | 5/39 | 176/18670 | 3.12E-05 | 0.000388659 | 0.000171184 | PTGS2/BCL2/TP53/MAPK14/MAPK8 | 5 |
| BP | GO:0032388 | positive regulation of intracellular transport | 6/39 | 229/18670 | 7.42E-06 | 0.000122665 | 5.40E-05 | PTGS2/BCL2/TP53/MAPK14/IL4R/MAPK8 | 6 |
| BP | GO:1903039 | positive regulation of leukocyte cell-cell adhesion | 6/39 | 218/18670 | 5.59E-06 | 9.99E-05 | 4.40E-05 | RELA/IL6ST/AKT1/IL4R/ICAM1/VCAM1 | 6 |
| BP | GO:1901030 | positive regulation of mitochondrial outer membrane permeabilization involved in apoptotic signaling pathway | 3/39 | 35/18670 | 5.27E-05 | 0.000571626 | 0.000251772 | BCL2/TP53/MAPK8 | 3 |
| BP | GO:0010822 | positive regulation of mitochondrion organization | 6/39 | 116/18670 | 1.39E-07 | 4.64E-06 | 2.04E-06 | MMP9/BCL2/TP53/HIF1A/KDR/MAPK8 | 6 |
| BP | GO:0045639 | positive regulation of myeloid cell differentiation | 4/39 | 91/18670 | 3.81E-05 | 0.00045161 | 0.000198911 | HIF1A/JUN/PRKCA/MAPK14 | 4 |
| BP | GO:0010831 | positive regulation of myotube differentiation | 3/39 | 38/18670 | 6.76E-05 | 0.000693859 | 0.000305609 | BCL2/MAPK14/IL4R | 3 |
| BP | GO:0045429 | positive regulation of nitric oxide biosynthetic process | 3/39 | 43/18670 | 9.82E-05 | 0.000950609 | 0.000418693 | PTGS2/AKT1/ICAM1 | 3 |
| BP | GO:1902895 | positive regulation of pri-miRNA transcription by RNA polymerase II | 4/39 | 31/18670 | 4.91E-07 | 1.32E-05 | 5.79E-06 | RELA/TP53/HIF1A/JUN | 4 |
| BP | GO:1900740 | positive regulation of protein insertion into mitochondrial membrane involved in apoptotic signaling pathway | 3/39 | 26/18670 | 2.12E-05 | 0.000286933 | 0.000126379 | BCL2/TP53/MAPK8 | 3 |
| BP | GO:0051222 | positive regulation of protein transport | 7/39 | 440/18670 | 3.08E-05 | 0.000385704 | 0.000169883 | PTGS2/BCL2/TP53/HIF1A/MAPK14/IL4R/MAPK8 | 7 |
| BP | GO:2000379 | positive regulation of reactive oxygen species metabolic process | 5/39 | 102/18670 | 2.19E-06 | 4.73E-05 | 2.09E-05 | PTGS2/AKT1/TP53/MAPK14/ICAM1 | 5 |
| BP | GO:0062013 | positive regulation of small molecule metabolic process | 5/39 | 141/18670 | 1.07E-05 | 0.000167093 | 7.36E-05 | PTGS2/AKT1/HIF1A/NOS2/PPARG | 5 |
| BP | GO:0048661 | positive regulation of smooth muscle cell proliferation | 6/39 | 101/18670 | 6.09E-08 | 2.46E-06 | 1.08E-06 | PTGS2/MMP2/MMP9/AKT1/JUN/HMOX1 | 6 |
| BP | GO:1901522 | positive regulation of transcription from RNA polymerase II promoter involved in cellular response to chemical stimulus | 3/39 | 22/18670 | 1.26E-05 | 0.000187217 | 8.25E-05 | RELA/TP53/HIF1A | 3 |
| BP | GO:0010575 | positive regulation of vascular endothelial growth factor production | 3/39 | 29/18670 | 2.97E-05 | 0.000378622 | 0.000166763 | PTGS2/IL6ST/HIF1A | 3 |
| BP | GO:1904018 | positive regulation of vasculature development | 6/39 | 230/18670 | 7.60E-06 | 0.000124921 | 5.50E-05 | PTGS2/CXCL8/HIF1A/PRKCA/KDR/HMOX1 | 6 |
| BP | GO:0045907 | positive regulation of vasoconstriction | 3/39 | 32/18670 | 4.01E-05 | 0.00046807 | 0.00020616 | PTGS2/AKT1/ICAM1 | 3 |
| BP | GO:0061614 | pri-miRNA transcription by RNA polymerase II | 5/39 | 47/18670 | 4.39E-08 | 2.04E-06 | 8.98E-07 | RELA/TP53/HIF1A/JUN/PPARG | 5 |
| BP | GO:0061919 | process utilizing autophagic mechanism | 8/39 | 496/18670 | 6.99E-06 | 0.000117139 | 5.16E-05 | AKT1/BCL2/CASP3/TP53/HIF1A/KDR/MAPK8/HMOX1 | 8 |
| BP | GO:0002532 | production of molecular mediator involved in inflammatory response | 4/39 | 72/18670 | 1.51E-05 | 0.000218841 | 9.64E-05 | NOS2/MAPK14/IL4R/ALOX5 | 4 |
| BP | GO:0017038 | protein import | 5/39 | 192/18670 | 4.73E-05 | 0.000530014 | 0.000233443 | PTGS2/NFKBIA/AKT1/TP53/MAPK14 | 5 |
| BP | GO:0006606 | protein import into nucleus | 5/39 | 143/18670 | 1.15E-05 | 0.000174504 | 7.69E-05 | PTGS2/NFKBIA/AKT1/TP53/MAPK14 | 5 |
| BP | GO:0001844 | protein insertion into mitochondrial membrane involved in apoptotic signaling pathway | 3/39 | 30/18670 | 3.29E-05 | 0.000401711 | 0.000176932 | BCL2/TP53/MAPK8 | 3 |
| BP | GO:2001057 | reactive nitrogen species metabolic process | 4/39 | 85/18670 | 2.91E-05 | 0.000373994 | 0.000164725 | PTGS2/AKT1/NOS2/ICAM1 | 4 |
| BP | GO:1903409 | reactive oxygen species biosynthetic process | 6/39 | 122/18670 | 1.88E-07 | 6.10E-06 | 2.69E-06 | PTGS2/AKT1/MPO/NOS2/CYP1A1/ICAM1 | 6 |
| BP | GO:0072593 | reactive oxygen species metabolic process | 11/39 | 284/18670 | 9.56E-12 | 1.81E-09 | 7.98E-10 | PTGS2/AKT1/BCL2/TP53/HIF1A/MPO/NOS2/MAPK14/NCF1/CYP1A1/ICAM1 | 11 |
| BP | GO:0045765 | regulation of angiogenesis | 7/39 | 383/18670 | 1.26E-05 | 0.000187217 | 8.25E-05 | PTGS2/CXCL8/HIF1A/PRKCA/PPARG/KDR/HMOX1 | 7 |
| BP | GO:2001233 | regulation of apoptotic signaling pathway | 11/39 | 406/18670 | 4.36E-10 | 4.88E-08 | 2.15E-08 | PTGS2/AR/RELA/MMP9/AKT1/BCL2/TP53/HIF1A/MAPK8/HMOX1/ICAM1 | 11 |
| BP | GO:0010506 | regulation of autophagy | 8/39 | 328/18670 | 3.19E-07 | 9.14E-06 | 4.03E-06 | AKT1/BCL2/CASP3/TP53/HIF1A/KDR/MAPK8/HMOX1 | 8 |
| BP | GO:0051098 | regulation of binding | 9/39 | 373/18670 | 5.75E-08 | 2.36E-06 | 1.04E-06 | MMP9/NFKBIA/AKT1/BCL2/JUN/PON1/PPARG/MAPK8/HMOX1 | 9 |
| BP | GO:0008217 | regulation of blood pressure | 6/39 | 182/18670 | 1.97E-06 | 4.42E-05 | 1.95E-05 | PTGS2/AR/PTGS1/NOS2/PPARG/HMOX1 | 6 |
| BP | GO:0043535 | regulation of blood vessel endothelial cell migration | 7/39 | 156/18670 | 3.05E-08 | 1.54E-06 | 6.76E-07 | PTGS2/AKT1/HIF1A/PRKCA/PPARG/KDR/HMOX1 | 7 |
| BP | GO:0022407 | regulation of cell-cell adhesion | 9/39 | 402/18670 | 1.09E-07 | 3.94E-06 | 1.74E-06 | RELA/IL6ST/AKT1/CASP3/PRKCA/MAPK14/IL4R/ICAM1/VCAM1 | 9 |
| BP | GO:0031279 | regulation of cyclase activity | 3/39 | 43/18670 | 9.82E-05 | 0.000950609 | 0.000418693 | NOS2/MAPK14/MAPK8 | 3 |
| BP | GO:0051101 | regulation of DNA binding | 6/39 | 124/18670 | 2.07E-07 | 6.46E-06 | 2.85E-06 | MMP9/NFKBIA/JUN/PPARG/MAPK8/HMOX1 | 6 |
| BP | GO:0051090 | regulation of DNA-binding transcription factor activity | 13/39 | 432/18670 | 2.14E-12 | 4.40E-10 | 1.94E-10 | AR/ESR1/RELA/NFKBIA/AKT1/JUN/PPARG/ESR2/MAPK14/IKBKB/MAPK8/HMOX1/ICAM1 | 13 |
| BP | GO:0043620 | regulation of DNA-templated transcription in response to stress | 5/39 | 127/18670 | 6.44E-06 | 0.00011169 | 4.92E-05 | RELA/TP53/HIF1A/JUN/HMOX1 | 5 |
| BP | GO:2000142 | regulation of DNA-templated transcription, initiation | 3/39 | 37/18670 | 6.23E-05 | 0.000653622 | 0.000287886 | ESR1/TP53/JUN | 3 |
| BP | GO:0010594 | regulation of endothelial cell migration | 7/39 | 229/18670 | 4.20E-07 | 1.14E-05 | 5.01E-06 | PTGS2/AKT1/HIF1A/PRKCA/PPARG/KDR/HMOX1 | 7 |
| BP | GO:0001936 | regulation of endothelial cell proliferation | 7/39 | 176/18670 | 7.00E-08 | 2.74E-06 | 1.21E-06 | AKT1/HIF1A/JUN/PRKCA/PPARG/KDR/HMOX1 | 7 |
| BP | GO:0010632 | regulation of epithelial cell migration | 9/39 | 291/18670 | 6.75E-09 | 4.50E-07 | 1.98E-07 | PTGS2/MMP9/AKT1/HIF1A/JUN/PRKCA/PPARG/KDR/HMOX1 | 9 |
| BP | GO:0050678 | regulation of epithelial cell proliferation | 9/39 | 378/18670 | 6.44E-08 | 2.56E-06 | 1.13E-06 | PGR/AR/AKT1/HIF1A/JUN/PRKCA/PPARG/KDR/HMOX1 | 9 |
| BP | GO:2001236 | regulation of extrinsic apoptotic signaling pathway | 6/39 | 155/18670 | 7.73E-07 | 1.96E-05 | 8.65E-06 | AR/RELA/AKT1/BCL2/HMOX1/ICAM1 | 6 |
| BP | GO:0048145 | regulation of fibroblast proliferation | 4/39 | 83/18670 | 2.65E-05 | 0.000345846 | 0.000152327 | ESR1/TP53/JUN/PPARG | 4 |
| BP | GO:0050727 | regulation of inflammatory response | 10/39 | 374/18670 | 3.50E-09 | 2.61E-07 | 1.15E-07 | PTGS2/ESR1/RELA/MMP9/NFKBIA/IL6ST/NOS2/PPARG/MAPK14/SELE | 10 |
| BP | GO:0032386 | regulation of intracellular transport | 7/39 | 370/18670 | 1.01E-05 | 0.000158542 | 6.98E-05 | PTGS2/BCL2/TP53/MAPK14/IL4R/MAPK8/HMOX1 | 7 |
| BP | GO:2001242 | regulation of intrinsic apoptotic signaling pathway | 6/39 | 165/18670 | 1.11E-06 | 2.72E-05 | 1.20E-05 | PTGS2/MMP9/AKT1/BCL2/TP53/HIF1A | 6 |
| BP | GO:1903037 | regulation of leukocyte cell-cell adhesion | 7/39 | 304/18670 | 2.79E-06 | 5.72E-05 | 2.52E-05 | RELA/IL6ST/AKT1/CASP3/IL4R/ICAM1/VCAM1 | 7 |
| BP | GO:0002685 | regulation of leukocyte migration | 5/39 | 196/18670 | 5.22E-05 | 0.000571626 | 0.000251772 | CXCL8/AKT1/MAPK14/HMOX1/ICAM1 | 5 |
| BP | GO:0070663 | regulation of leukocyte proliferation | 5/39 | 222/18670 | 9.41E-05 | 0.000927548 | 0.000408536 | IL6ST/BCL2/CASP3/AHR/VCAM1 | 5 |
| BP | GO:0051249 | regulation of lymphocyte activation | 7/39 | 485/18670 | 5.72E-05 | 0.00061457 | 0.000270686 | IL6ST/AKT1/BCL2/CASP3/AHR/IL4R/VCAM1 | 7 |
| BP | GO:0050670 | regulation of lymphocyte proliferation | 5/39 | 208/18670 | 6.92E-05 | 0.000702535 | 0.00030943 | IL6ST/BCL2/CASP3/AHR/VCAM1 | 5 |
| BP | GO:0016241 | regulation of macroautophagy | 7/39 | 171/18670 | 5.74E-08 | 2.36E-06 | 1.04E-06 | AKT1/CASP3/TP53/HIF1A/KDR/MAPK8/HMOX1 | 7 |
| BP | GO:0010821 | regulation of mitochondrion organization | 7/39 | 179/18670 | 7.86E-08 | 2.98E-06 | 1.31E-06 | MMP9/AKT1/BCL2/TP53/HIF1A/KDR/MAPK8 | 7 |
| BP | GO:0032944 | regulation of mononuclear cell proliferation | 5/39 | 209/18670 | 7.08E-05 | 0.000714819 | 0.000314841 | IL6ST/BCL2/CASP3/AHR/VCAM1 | 5 |
| BP | GO:0043523 | regulation of neuron apoptotic process | 6/39 | 210/18670 | 4.51E-06 | 8.36E-05 | 3.68E-05 | BCL2/CASP3/TP53/HIF1A/JUN/HMOX1 | 6 |
| BP | GO:1901214 | regulation of neuron death | 7/39 | 313/18670 | 3.38E-06 | 6.77E-05 | 2.98E-05 | AKT1/BCL2/CASP3/TP53/HIF1A/JUN/HMOX1 | 7 |
| BP | GO:1902893 | regulation of pri-miRNA transcription by RNA polymerase II | 4/39 | 41/18670 | 1.56E-06 | 3.65E-05 | 1.61E-05 | RELA/TP53/HIF1A/JUN | 4 |
| BP | GO:1900739 | regulation of protein insertion into mitochondrial membrane involved in apoptotic signaling pathway | 3/39 | 26/18670 | 2.12E-05 | 0.000286933 | 0.000126379 | BCL2/TP53/MAPK8 | 3 |
| BP | GO:1905475 | regulation of protein localization to membrane | 5/39 | 187/18670 | 4.17E-05 | 0.000482744 | 0.000212623 | AR/AKT1/BCL2/TP53/MAPK8 | 5 |
| BP | GO:2000377 | regulation of reactive oxygen species metabolic process | 7/39 | 195/18670 | 1.41E-07 | 4.64E-06 | 2.04E-06 | PTGS2/AKT1/BCL2/TP53/HIF1A/MAPK14/ICAM1 | 7 |
| BP | GO:0048660 | regulation of smooth muscle cell proliferation | 7/39 | 169/18670 | 5.30E-08 | 2.36E-06 | 1.04E-06 | PTGS2/MMP2/MMP9/AKT1/JUN/PPARG/HMOX1 | 7 |
| BP | GO:0010574 | regulation of vascular endothelial growth factor production | 3/39 | 34/18670 | 4.82E-05 | 0.000535174 | 0.000235716 | PTGS2/IL6ST/HIF1A | 3 |
| BP | GO:1904705 | regulation of vascular smooth muscle cell proliferation | 5/39 | 85/18670 | 8.85E-07 | 2.20E-05 | 9.70E-06 | MMP2/MMP9/JUN/PPARG/HMOX1 | 5 |
| BP | GO:1901342 | regulation of vasculature development | 7/39 | 422/18670 | 2.36E-05 | 0.000316136 | 0.000139242 | PTGS2/CXCL8/HIF1A/PRKCA/PPARG/KDR/HMOX1 | 7 |
| BP | GO:0001836 | release of cytochrome c from mitochondria | 5/39 | 59/18670 | 1.41E-07 | 4.64E-06 | 2.04E-06 | MMP9/AKT1/BCL2/TP53/JUN | 5 |
| BP | GO:0048608 | reproductive structure development | 11/39 | 431/18670 | 8.19E-10 | 8.35E-08 | 3.68E-08 | PTGS2/PGR/AR/ESR1/AKT1/BCL2/CASP3/HIF1A/PPARG/MAPK14/ICAM1 | 11 |
| BP | GO:0061458 | reproductive system development | 11/39 | 434/18670 | 8.81E-10 | 8.35E-08 | 3.68E-08 | PTGS2/PGR/AR/ESR1/AKT1/BCL2/CASP3/HIF1A/PPARG/MAPK14/ICAM1 | 11 |
| BP | GO:0001101 | response to acid chemical | 9/39 | 343/18670 | 2.80E-08 | 1.44E-06 | 6.34E-07 | PTGS2/RELA/MMP2/AKT1/CASP3/PON1/PPARG/KDR/ICAM1 | 9 |
| BP | GO:0043279 | response to alkaloid | 4/39 | 112/18670 | 8.58E-05 | 0.00085293 | 0.000375671 | RELA/CASP3/PPARG/ICAM1 | 4 |
| BP | GO:0043200 | response to amino acid | 4/39 | 113/18670 | 8.89E-05 | 0.000879361 | 0.000387313 | RELA/MMP2/CASP3/ICAM1 | 4 |
| BP | GO:1904645 | response to amyloid-beta | 4/39 | 54/18670 | 4.77E-06 | 8.64E-05 | 3.81E-05 | MMP2/MMP9/ICAM1/VCAM1 | 4 |
| BP | GO:0046677 | response to antibiotic | 10/39 | 327/18670 | 9.60E-10 | 8.76E-08 | 3.86E-08 | RELA/BCL2/CASP3/TP53/AHR/JUN/HMOX1/CYP1A1/ICAM1/VCAM1 | 10 |
| BP | GO:0046686 | response to cadmium ion | 6/39 | 63/18670 | 3.46E-09 | 2.61E-07 | 1.15E-07 | MMP9/AKT1/JUN/NCF1/MAPK8/HMOX1 | 6 |
| BP | GO:0036293 | response to decreased oxygen levels | 13/39 | 370/18670 | 3.01E-13 | 9.27E-11 | 4.08E-11 | PTGS2/MMP2/AKT1/BCL2/CASP3/TP53/HIF1A/PLAU/NOS2/HMOX1/CYP1A1/ICAM1/VCAM1 | 13 |
| BP | GO:0043627 | response to estrogen | 4/39 | 73/18670 | 1.59E-05 | 0.000228492 | 0.000100639 | ESR1/PPARG/IL4R/HMOX1 | 4 |
| BP | GO:0070542 | response to fatty acid | 4/39 | 86/18670 | 3.05E-05 | 0.000383657 | 0.000168981 | PTGS2/AKT1/PON1/PPARG | 4 |
| BP | GO:0032094 | response to food | 3/39 | 38/18670 | 6.76E-05 | 0.000693859 | 0.000305609 | AKT1/MPO/CYP1A1 | 3 |
| BP | GO:0042542 | response to hydrogen peroxide | 5/39 | 146/18670 | 1.27E-05 | 0.000187217 | 8.25E-05 | RELA/BCL2/CASP3/JUN/HMOX1 | 5 |
| BP | GO:0001666 | response to hypoxia | 13/39 | 359/18670 | 2.05E-13 | 8.02E-11 | 3.53E-11 | PTGS2/MMP2/AKT1/BCL2/CASP3/TP53/HIF1A/PLAU/NOS2/HMOX1/CYP1A1/ICAM1/VCAM1 | 13 |
| BP | GO:0034341 | response to interferon-gamma | 5/39 | 199/18670 | 5.61E-05 | 0.00060635 | 0.000267066 | TP53/NOS2/PPARG/ICAM1/VCAM1 | 5 |
| BP | GO:0070555 | response to interleukin-1 | 8/39 | 207/18670 | 9.14E-09 | 5.77E-07 | 2.54E-07 | RELA/NFKBIA/CXCL8/HIF1A/PRKCA/IKBKB/ICAM1/SELE | 8 |
| BP | GO:0010212 | response to ionizing radiation | 6/39 | 147/18670 | 5.66E-07 | 1.50E-05 | 6.61E-06 | BCL2/CASP3/TP53/MAPK14/ICAM1/VCAM1 | 6 |
| BP | GO:0010039 | response to iron ion | 4/39 | 33/18670 | 6.37E-07 | 1.67E-05 | 7.35E-06 | BCL2/HIF1A/HMOX1/CYP1A1 | 4 |
| BP | GO:1901654 | response to ketone | 6/39 | 193/18670 | 2.77E-06 | 5.72E-05 | 2.52E-05 | AR/RELA/AKT1/AHR/PPARG/ICAM1 | 6 |
| BP | GO:0009416 | response to light stimulus | 8/39 | 314/18670 | 2.29E-07 | 6.88E-06 | 3.03E-06 | PTGS2/RELA/AKT1/BCL2/CASP3/TP53/HIF1A/MAPK8 | 8 |
| BP | GO:0032496 | response to lipopolysaccharide | 16/39 | 330/18670 | 1.65E-18 | 3.77E-15 | 1.66E-15 | PTGS2/RELA/NFKBIA/CXCL8/AKT1/CASP3/MPO/JUN/PRKCA/NOS2/MAPK14/MAPK8/CYP1A1/ICAM1/SELE/VCAM1 | 16 |
| BP | GO:0009612 | response to mechanical stimulus | 9/39 | 210/18670 | 3.84E-10 | 4.73E-08 | 2.08E-08 | PTGS2/RELA/NFKBIA/AKT1/MPO/JUN/PPARG/MAPK14/MAPK8 | 9 |
| BP | GO:0010038 | response to metal ion | 13/39 | 362/18670 | 2.28E-13 | 8.02E-11 | 3.53E-11 | PTGS2/MMP9/AKT1/BCL2/CASP3/HIF1A/JUN/NCF1/MAPK8/HMOX1/CYP1A1/ICAM1/VCAM1 | 13 |
| BP | GO:0002237 | response to molecule of bacterial origin | 16/39 | 343/18670 | 3.06E-18 | 3.77E-15 | 1.66E-15 | PTGS2/RELA/NFKBIA/CXCL8/AKT1/CASP3/MPO/JUN/PRKCA/NOS2/MAPK14/MAPK8/CYP1A1/ICAM1/SELE/VCAM1 | 16 |
| BP | GO:0032495 | response to muramyl dipeptide | 3/39 | 20/18670 | 9.37E-06 | 0.00014954 | 6.59E-05 | RELA/NFKBIA/MAPK14 | 3 |
| BP | GO:0035994 | response to muscle stretch | 4/39 | 18/18670 | 4.87E-08 | 2.22E-06 | 9.79E-07 | RELA/NFKBIA/JUN/MAPK14 | 4 |
| BP | GO:0035094 | response to nicotine | 5/39 | 49/18670 | 5.44E-08 | 2.36E-06 | 1.04E-06 | RELA/BCL2/CASP3/HMOX1/VCAM1 | 5 |
| BP | GO:0007584 | response to nutrient | 6/39 | 219/18670 | 5.74E-06 | 0.000101819 | 4.48E-05 | PTGS2/RELA/PPARG/HMOX1/CYP1A1/VCAM1 | 6 |
| BP | GO:0031667 | response to nutrient levels | 14/39 | 499/18670 | 6.48E-13 | 1.68E-10 | 7.42E-11 | PTGS2/RELA/AKT1/BCL2/TP53/MPO/JUN/PON1/PPARG/MAPK8/HMOX1/CYP1A1/ICAM1/VCAM1 | 14 |
| BP | GO:0006979 | response to oxidative stress | 15/39 | 451/18670 | 6.54E-15 | 4.03E-12 | 1.77E-12 | PTGS2/RELA/MMP2/MMP9/PTGS1/AKT1/BCL2/CASP3/TP53/HIF1A/MPO/JUN/NCF1/MAPK8/HMOX1 | 15 |
| BP | GO:0070482 | response to oxygen levels | 14/39 | 394/18670 | 2.58E-14 | 1.27E-11 | 5.60E-12 | PTGS2/MMP2/AKT1/BCL2/CASP3/TP53/HIF1A/PLAU/NOS2/PPARG/HMOX1/CYP1A1/ICAM1/VCAM1 | 14 |
| BP | GO:0014074 | response to purine-containing compound | 5/39 | 149/18670 | 1.40E-05 | 0.000204162 | 8.99E-05 | PTGS2/RELA/AHR/JUN/PPARG | 5 |
| BP | GO:0009314 | response to radiation | 12/39 | 448/18670 | 6.85E-11 | 9.92E-09 | 4.37E-09 | PTGS2/RELA/AKT1/BCL2/CASP3/TP53/HIF1A/JUN/MAPK14/MAPK8/ICAM1/VCAM1 | 12 |
| BP | GO:0000302 | response to reactive oxygen species | 11/39 | 232/18670 | 1.06E-12 | 2.38E-10 | 1.05E-10 | RELA/MMP2/MMP9/AKT1/BCL2/CASP3/MPO/JUN/NCF1/MAPK8/HMOX1 | 11 |
| BP | GO:0042594 | response to starvation | 5/39 | 191/18670 | 4.62E-05 | 0.000521739 | 0.000229799 | BCL2/TP53/JUN/PPARG/MAPK8 | 5 |
| BP | GO:0048545 | response to steroid hormone | 10/39 | 383/18670 | 4.39E-09 | 3.09E-07 | 1.36E-07 | PTGS2/PGR/AR/ESR1/RELA/BCL2/CASP3/PPARG/ESR2/ICAM1 | 10 |
| BP | GO:0034612 | response to tumor necrosis factor | 11/39 | 312/18670 | 2.63E-11 | 4.63E-09 | 2.04E-09 | PTGS2/RELA/NFKBIA/CXCL8/AKT1/CASP3/MAPK14/IKBKB/ICAM1/SELE/VCAM1 | 11 |
| BP | GO:0009411 | response to UV | 7/39 | 141/18670 | 1.52E-08 | 8.49E-07 | 3.74E-07 | PTGS2/RELA/AKT1/BCL2/CASP3/TP53/MAPK8 | 7 |
| BP | GO:0033273 | response to vitamin | 4/39 | 93/18670 | 4.15E-05 | 0.0004826 | 0.00021256 | PTGS2/RELA/PPARG/CYP1A1 | 4 |
| BP | GO:0009410 | response to xenobiotic stimulus | 7/39 | 292/18670 | 2.13E-06 | 4.65E-05 | 2.05E-05 | RELA/GSTM1/PTGS1/AHR/PPARG/CYP1A1/ICAM1 | 7 |
| BP | GO:0048511 | rhythmic process | 9/39 | 295/18670 | 7.60E-09 | 4.93E-07 | 2.17E-07 | PGR/ESR1/CASP3/TP53/AHR/JUN/NOS2/PPARG/MAPK8 | 9 |
| BP | GO:0019932 | second-messenger-mediated signaling | 7/39 | 439/18670 | 3.04E-05 | 0.000383657 | 0.000168981 | CXCL8/AHR/PRKCA/NOS2/KDR/SELE/VCAM1 | 7 |
| BP | GO:0007548 | sex differentiation | 6/39 | 270/18670 | 1.89E-05 | 0.000263264 | 0.000115954 | PGR/AR/ESR1/BCL2/CASP3/ICAM1 | 6 |
| BP | GO:0048659 | smooth muscle cell proliferation | 7/39 | 171/18670 | 5.74E-08 | 2.36E-06 | 1.04E-06 | PTGS2/MMP2/MMP9/AKT1/JUN/PPARG/HMOX1 | 7 |
| BP | GO:0043401 | steroid hormone mediated signaling pathway | 5/39 | 182/18670 | 3.67E-05 | 0.00043856 | 0.000193163 | PGR/AR/ESR1/PPARG/ESR2 | 5 |
| BP | GO:0042110 | T cell activation | 8/39 | 464/18670 | 4.28E-06 | 8.00E-05 | 3.52E-05 | IL6ST/AKT1/BCL2/CASP3/TP53/IL4R/ICAM1/VCAM1 | 8 |
| BP | GO:0043029 | T cell homeostasis | 3/39 | 37/18670 | 6.23E-05 | 0.000653622 | 0.000287886 | AKT1/BCL2/CASP3 | 3 |
| BP | GO:0090130 | tissue migration | 9/39 | 361/18670 | 4.34E-08 | 2.04E-06 | 8.98E-07 | PTGS2/MMP9/AKT1/HIF1A/JUN/PRKCA/PPARG/KDR/HMOX1 | 9 |
| BP | GO:0006367 | transcription initiation from RNA polymerase II promoter | 6/39 | 188/18670 | 2.38E-06 | 5.06E-05 | 2.23E-05 | PGR/AR/ESR1/TP53/PPARG/ESR2 | 6 |
| BP | GO:0033559 | unsaturated fatty acid metabolic process | 4/39 | 110/18670 | 8.00E-05 | 0.000801674 | 0.000353095 | PTGS2/PTGS1/CYP1A1/ALOX5 | 4 |
| BP | GO:0010573 | vascular endothelial growth factor production | 3/39 | 36/18670 | 5.74E-05 | 0.00061457 | 0.000270686 | PTGS2/IL6ST/HIF1A | 3 |
| BP | GO:0048010 | vascular endothelial growth factor receptor signaling pathway | 4/39 | 96/18670 | 4.70E-05 | 0.000529148 | 0.000233062 | HIF1A/MAPK14/NCF1/KDR | 4 |
| BP | GO:1990874 | vascular smooth muscle cell proliferation | 5/39 | 85/18670 | 8.85E-07 | 2.20E-05 | 9.70E-06 | MMP2/MMP9/JUN/PPARG/HMOX1 | 5 |

| Supplementary table 2. Enrichment analysis of KEGG | | | | | | | | |
| --- | --- | --- | --- | --- | --- | --- | --- | --- |
| ID | Description | GeneRatio | BgRatio | pvalue | p.adjust | qvalue | geneID | Count |
| hsa04920 | Adipocytokine signaling pathway | 5/38 | 69/7220 | 2.71E-05 | 0.000130279 | 5.49E-05 | RELA/NFKBIA/AKT1/IKBKB/MAPK8 | 5 |
| hsa04261 | Adrenergic signaling in cardiomyocytes | 4/38 | 150/7220 | 0.007632374 | 0.016817096 | 0.007080882 | AKT1/BCL2/PRKCA/MAPK14 | 4 |
| hsa04933 | AGE-RAGE signaling pathway in diabetic complications | 13/38 | 100/7220 | 1.26E-15 | 1.64E-13 | 6.91E-14 | RELA/MMP2/CXCL8/AKT1/BCL2/CASP3/JUN/PRKCA/MAPK14/MAPK8/ICAM1/SELE/VCAM1 | 13 |
| hsa04936 | Alcoholic liver disease | 8/38 | 142/7220 | 5.46E-07 | 4.44E-06 | 1.87E-06 | RELA/NFKBIA/CXCL8/AKT1/CASP3/MAPK14/IKBKB/MAPK8 | 8 |
| hsa05010 | Alzheimer disease | 7/38 | 384/7220 | 0.003432475 | 0.009106566 | 0.003834343 | PTGS2/RELA/AKT1/CASP3/NOS2/IKBKB/MAPK8 | 7 |
| hsa01523 | Antifolate resistance | 2/38 | 31/7220 | 0.011393676 | 0.023889966 | 0.010058933 | RELA/IKBKB | 2 |
| hsa04210 | Apoptosis | 9/38 | 136/7220 | 2.35E-08 | 3.05E-07 | 1.29E-07 | RELA/NFKBIA/AKT1/BCL2/CASP3/TP53/JUN/IKBKB/MAPK8 | 9 |
| hsa04215 | Apoptosis - multiple species | 3/38 | 32/7220 | 0.000600527 | 0.002001757 | 0.000842845 | BCL2/CASP3/MAPK8 | 3 |
| hsa00590 | Arachidonic acid metabolism | 3/38 | 61/7220 | 0.003922974 | 0.010199732 | 0.004294624 | PTGS2/PTGS1/ALOX5 | 3 |
| hsa04140 | Autophagy - animal | 4/38 | 141/7220 | 0.006147339 | 0.014270607 | 0.006008677 | AKT1/BCL2/HIF1A/MAPK8 | 4 |
| hsa04662 | B cell receptor signaling pathway | 5/38 | 82/7220 | 6.25E-05 | 0.000270875 | 0.000114053 | RELA/NFKBIA/AKT1/JUN/IKBKB | 5 |
| hsa04024 | cAMP signaling pathway | 5/38 | 219/7220 | 0.005443857 | 0.012867297 | 0.005417809 | RELA/NFKBIA/AKT1/JUN/MAPK8 | 5 |
| hsa04514 | Cell adhesion molecules | 3/38 | 149/7220 | 0.04294408 | 0.07345698 | 0.030929255 | ICAM1/SELE/VCAM1 | 3 |
| hsa04218 | Cellular senescence | 5/38 | 156/7220 | 0.001246222 | 0.00368202 | 0.001550324 | RELA/CXCL8/AKT1/TP53/MAPK14 | 5 |
| hsa04062 | Chemokine signaling pathway | 6/38 | 192/7220 | 0.000444104 | 0.001560367 | 0.000656997 | RELA/NFKBIA/CXCL8/AKT1/NCF1/IKBKB | 6 |
| hsa04725 | Cholinergic synapse | 3/38 | 113/7220 | 0.02116489 | 0.04168842 | 0.017553019 | AKT1/BCL2/PRKCA | 3 |
| hsa04625 | C-type lectin receptor signaling pathway | 8/38 | 104/7220 | 4.83E-08 | 5.23E-07 | 2.20E-07 | PTGS2/RELA/NFKBIA/AKT1/JUN/MAPK14/IKBKB/MAPK8 | 8 |
| hsa04623 | Cytosolic DNA-sensing pathway | 3/38 | 63/7220 | 0.004297399 | 0.010954153 | 0.004612275 | RELA/NFKBIA/IKBKB | 3 |
| hsa04728 | Dopaminergic synapse | 4/38 | 132/7220 | 0.004869416 | 0.011722667 | 0.00493586 | AKT1/PRKCA/MAPK14/MAPK8 | 4 |
| hsa01521 | EGFR tyrosine kinase inhibitor resistance | 4/38 | 79/7220 | 0.000738904 | 0.00240144 | 0.001011132 | AKT1/BCL2/PRKCA/KDR | 4 |
| hsa04961 | Endocrine and other factor-regulated calcium reabsorption | 2/38 | 53/7220 | 0.031408286 | 0.056709406 | 0.023877645 | ESR1/PRKCA | 2 |
| hsa01522 | Endocrine resistance | 10/38 | 98/7220 | 4.59E-11 | 1.19E-09 | 5.03E-10 | ESR1/MMP2/MMP9/AKT1/BCL2/TP53/JUN/ESR2/MAPK14/MAPK8 | 10 |
| hsa04012 | ErbB signaling pathway | 4/38 | 85/7220 | 0.000973531 | 0.003086806 | 0.001299708 | AKT1/JUN/PRKCA/MAPK8 | 4 |
| hsa04915 | Estrogen signaling pathway | 8/38 | 138/7220 | 4.38E-07 | 3.80E-06 | 1.60E-06 | PGR/ESR1/MMP2/MMP9/AKT1/BCL2/JUN/ESR2 | 8 |
| hsa04664 | Fc epsilon RI signaling pathway | 5/38 | 68/7220 | 2.52E-05 | 0.000125966 | 5.30E-05 | AKT1/PRKCA/MAPK14/MAPK8/ALOX5 | 5 |
| hsa04666 | Fc gamma R-mediated phagocytosis | 3/38 | 97/7220 | 0.014115657 | 0.029127545 | 0.01226423 | AKT1/PRKCA/NCF1 | 3 |
| hsa04216 | Ferroptosis | 2/38 | 41/7220 | 0.019440885 | 0.038881771 | 0.016371272 | TP53/HMOX1 | 2 |
| hsa04510 | Focal adhesion | 6/38 | 201/7220 | 0.000566784 | 0.001938998 | 0.00081642 | AKT1/BCL2/JUN/PRKCA/KDR/MAPK8 | 6 |
| hsa04068 | FoxO signaling pathway | 4/38 | 131/7220 | 0.004739598 | 0.011625429 | 0.004894917 | AKT1/MAPK14/IKBKB/MAPK8 | 4 |
| hsa04929 | GnRH secretion | 3/38 | 64/7220 | 0.004492486 | 0.011231216 | 0.004728933 | AKT1/PRKCA/ESR2 | 3 |
| hsa04912 | GnRH signaling pathway | 5/38 | 93/7220 | 0.000114165 | 0.000478754 | 0.000201581 | MMP2/JUN/PRKCA/MAPK14/MAPK8 | 5 |
| hsa04935 | Growth hormone synthesis, secretion and action | 4/38 | 119/7220 | 0.003360099 | 0.009100269 | 0.003831692 | AKT1/PRKCA/MAPK14/MAPK8 | 4 |
| hsa04066 | HIF-1 signaling pathway | 7/38 | 109/7220 | 1.26E-06 | 9.13E-06 | 3.84E-06 | RELA/AKT1/BCL2/HIF1A/PRKCA/NOS2/HMOX1 | 7 |
| hsa04657 | IL-17 signaling pathway | 11/38 | 94/7220 | 9.03E-13 | 3.91E-11 | 1.65E-11 | PTGS2/RELA/MMP9/NFKBIA/CXCL8/CASP3/JUN/MAPK14/IKBKB/MAPK8/MMP1 | 11 |
| hsa04750 | Inflammatory mediator regulation of TRP channels | 3/38 | 98/7220 | 0.014509184 | 0.02947178 | 0.01240917 | PRKCA/MAPK14/MAPK8 | 3 |
| hsa04931 | Insulin resistance | 5/38 | 108/7220 | 0.000231226 | 0.000939357 | 0.000395519 | RELA/NFKBIA/AKT1/IKBKB/MAPK8 | 5 |
| hsa04910 | Insulin signaling pathway | 3/38 | 137/7220 | 0.034778513 | 0.061934338 | 0.026077616 | AKT1/IKBKB/MAPK8 | 3 |
| hsa04630 | JAK-STAT signaling pathway | 4/38 | 162/7220 | 0.009957543 | 0.021574676 | 0.009084074 | IL6ST/AKT1/BCL2/IL4R | 4 |
| hsa04670 | Leukocyte transendothelial migration | 7/38 | 114/7220 | 1.71E-06 | 1.06E-05 | 4.46E-06 | MMP2/MMP9/PRKCA/MAPK14/NCF1/ICAM1/VCAM1 | 7 |
| hsa04211 | Longevity regulating pathway | 4/38 | 89/7220 | 0.001156424 | 0.003579406 | 0.001507118 | RELA/AKT1/TP53/PPARG | 4 |
| hsa04010 | MAPK signaling pathway | 10/38 | 294/7220 | 1.85E-06 | 1.09E-05 | 4.59E-06 | RELA/AKT1/CASP3/TP53/JUN/PRKCA/MAPK14/KDR/IKBKB/MAPK8 | 10 |
| hsa04137 | Mitophagy - animal | 5/38 | 72/7220 | 3.33E-05 | 0.000154618 | 6.51E-05 | RELA/TP53/HIF1A/JUN/MAPK8 | 5 |
| hsa04150 | mTOR signaling pathway | 3/38 | 155/7220 | 0.047359516 | 0.078932527 | 0.033234748 | AKT1/PRKCA/IKBKB | 3 |
| hsa04650 | Natural killer cell mediated cytotoxicity | 3/38 | 131/7220 | 0.031033238 | 0.056709406 | 0.023877645 | CASP3/PRKCA/ICAM1 | 3 |
| hsa04722 | Neurotrophin signaling pathway | 9/38 | 119/7220 | 7.22E-09 | 1.17E-07 | 4.94E-08 | RELA/NFKBIA/AKT1/BCL2/TP53/JUN/MAPK14/IKBKB/MAPK8 | 9 |
| hsa04613 | Neutrophil extracellular trap formation | 6/38 | 190/7220 | 0.000419918 | 0.001516371 | 0.000638472 | RELA/AKT1/MPO/PRKCA/MAPK14/NCF1 | 6 |
| hsa04064 | NF-kappa B signaling pathway | 9/38 | 104/7220 | 2.17E-09 | 4.03E-08 | 1.70E-08 | PTGS2/RELA/NFKBIA/CXCL8/BCL2/PLAU/IKBKB/ICAM1/VCAM1 | 9 |
| hsa04621 | NOD-like receptor signaling pathway | 8/38 | 184/7220 | 3.88E-06 | 2.10E-05 | 8.86E-06 | RELA/NFKBIA/CXCL8/BCL2/JUN/MAPK14/IKBKB/MAPK8 | 8 |
| hsa04932 | Non-alcoholic fatty liver disease | 9/38 | 155/7220 | 7.35E-08 | 7.35E-07 | 3.10E-07 | RELA/CXCL8/AKT1/CASP3/JUN/PPARG/MAPK14/IKBKB/MAPK8 | 9 |
| hsa04114 | Oocyte meiosis | 3/38 | 131/7220 | 0.031033238 | 0.056709406 | 0.023877645 | PGR/AR/MAPK14 | 3 |
| hsa04380 | Osteoclast differentiation | 9/38 | 128/7220 | 1.38E-08 | 1.99E-07 | 8.38E-08 | RELA/NFKBIA/AKT1/JUN/PPARG/MAPK14/NCF1/IKBKB/MAPK8 | 9 |
| hsa04913 | Ovarian steroidogenesis | 3/38 | 51/7220 | 0.002353568 | 0.006651386 | 0.002800584 | PTGS2/CYP1A1/ALOX5 | 3 |
| hsa04921 | Oxytocin signaling pathway | 3/38 | 154/7220 | 0.0466084 | 0.078689507 | 0.033132424 | PTGS2/JUN/PRKCA | 3 |
| hsa04115 | p53 signaling pathway | 3/38 | 73/7220 | 0.006491991 | 0.014806295 | 0.006234229 | BCL2/CASP3/TP53 | 3 |
| hsa04072 | Phospholipase D signaling pathway | 3/38 | 148/7220 | 0.04222957 | 0.073197921 | 0.030820177 | CXCL8/AKT1/PRKCA | 3 |
| hsa04151 | PI3K-Akt signaling pathway | 8/38 | 354/7220 | 0.000413374 | 0.001516371 | 0.000638472 | RELA/AKT1/BCL2/TP53/PRKCA/IL4R/KDR/IKBKB | 8 |
| hsa04611 | Platelet activation | 3/38 | 124/7220 | 0.026951172 | 0.050777571 | 0.02138003 | PTGS1/AKT1/MAPK14 | 3 |
| hsa01524 | Platinum drug resistance | 5/38 | 73/7220 | 3.56E-05 | 0.000159652 | 6.72E-05 | GSTM1/AKT1/BCL2/CASP3/TP53 | 5 |
| hsa04914 | Progesterone-mediated oocyte maturation | 4/38 | 102/7220 | 0.001916732 | 0.005537227 | 0.002331464 | PGR/AKT1/MAPK14/MAPK8 | 4 |
| hsa04917 | Prolactin signaling pathway | 6/38 | 70/7220 | 1.44E-06 | 9.39E-06 | 3.95E-06 | ESR1/RELA/AKT1/ESR2/MAPK14/MAPK8 | 6 |
| hsa04015 | Rap1 signaling pathway | 4/38 | 210/7220 | 0.0237052 | 0.045995164 | 0.019366385 | AKT1/PRKCA/MAPK14/KDR | 4 |
| hsa04014 | Ras signaling pathway | 6/38 | 232/7220 | 0.001202091 | 0.003634227 | 0.001530201 | RELA/AKT1/PRKCA/KDR/IKBKB/MAPK8 | 6 |
| hsa04923 | Regulation of lipolysis in adipocytes | 3/38 | 57/7220 | 0.003235849 | 0.008950222 | 0.003768514 | PTGS2/PTGS1/AKT1 | 3 |
| hsa04926 | Relaxin signaling pathway | 11/38 | 129/7220 | 3.08E-11 | 1.00E-09 | 4.21E-10 | RELA/MMP2/MMP9/NFKBIA/AKT1/JUN/PRKCA/NOS2/MAPK14/MAPK8/MMP1 | 11 |
| hsa04723 | Retrograde endocannabinoid signaling | 4/38 | 148/7220 | 0.007283769 | 0.016325689 | 0.006873974 | PTGS2/PRKCA/MAPK14/MAPK8 | 4 |
| hsa04622 | RIG-I-like receptor signaling pathway | 6/38 | 70/7220 | 1.44E-06 | 9.39E-06 | 3.95E-06 | RELA/NFKBIA/CXCL8/MAPK14/IKBKB/MAPK8 | 6 |
| hsa04726 | Serotonergic synapse | 5/38 | 115/7220 | 0.000309964 | 0.001221069 | 0.000514134 | PTGS2/PTGS1/CASP3/PRKCA/ALOX5 | 5 |
| hsa04550 | Signaling pathways regulating pluripotency of stem cells | 3/38 | 143/7220 | 0.038749518 | 0.068073478 | 0.028662517 | IL6ST/AKT1/MAPK14 | 3 |
| hsa04071 | Sphingolipid signaling pathway | 7/38 | 119/7220 | 2.29E-06 | 1.29E-05 | 5.44E-06 | RELA/AKT1/BCL2/TP53/PRKCA/MAPK14/MAPK8 | 7 |
| hsa04660 | T cell receptor signaling pathway | 7/38 | 104/7220 | 9.18E-07 | 7.02E-06 | 2.96E-06 | RELA/NFKBIA/AKT1/JUN/MAPK14/IKBKB/MAPK8 | 7 |
| hsa04658 | Th1 and Th2 cell differentiation | 7/38 | 92/7220 | 3.96E-07 | 3.68E-06 | 1.55E-06 | RELA/NFKBIA/JUN/MAPK14/IL4R/IKBKB/MAPK8 | 7 |
| hsa04659 | Th17 cell differentiation | 10/38 | 108/7220 | 1.23E-10 | 2.66E-09 | 1.12E-09 | RELA/NFKBIA/IL6ST/HIF1A/AHR/JUN/MAPK14/IL4R/IKBKB/MAPK8 | 10 |
| hsa04919 | Thyroid hormone signaling pathway | 5/38 | 121/7220 | 0.000392389 | 0.001500311 | 0.00063171 | ESR1/AKT1/TP53/HIF1A/PRKCA | 5 |
| hsa04668 | TNF signaling pathway | 13/38 | 112/7220 | 5.80E-15 | 3.77E-13 | 1.59E-13 | PTGS2/RELA/MMP9/NFKBIA/AKT1/CASP3/JUN/MAPK14/IKBKB/MAPK8/ICAM1/SELE/VCAM1 | 13 |
| hsa04620 | Toll-like receptor signaling pathway | 8/38 | 104/7220 | 4.83E-08 | 5.23E-07 | 2.20E-07 | RELA/NFKBIA/CXCL8/AKT1/JUN/MAPK14/IKBKB/MAPK8 | 8 |
| hsa04930 | Type II diabetes mellitus | 2/38 | 46/7220 | 0.024138268 | 0.046146688 | 0.019430185 | IKBKB/MAPK8 | 2 |
| hsa04370 | VEGF signaling pathway | 5/38 | 59/7220 | 1.25E-05 | 6.51E-05 | 2.74E-05 | PTGS2/AKT1/PRKCA/MAPK14/KDR | 5 |
| hsa04310 | Wnt signaling pathway | 4/38 | 166/7220 | 0.01082477 | 0.023069181 | 0.009713339 | TP53/JUN/PRKCA/MAPK8 | 4 |
